# Planckian Power Spectral Densities from Human Calves during Posture Maintenance and Controlled Isometric Contractions

**DOI:** 10.1371/journal.pone.0131798

**Published:** 2015-07-27

**Authors:** J. E. Lugo, Rafael Doti, Jocelyn Faubert

**Affiliations:** Visual psychophysics and perception laboratory, School of Optometry, Université de Montréal, Montréal, Quebéc, Canada; National Institute of Genomic Medicine, MEXICO

## Abstract

**Background:**

The relationship between muscle anatomy and physiology and its corresponding electromyography activity (EMGA) is complex and not well understood. EMGA models may be broadly divided in stochastic and motor-unit-based models. For example, these models have successfully described many muscle physiological variables such as the value of the muscle fiber velocity and the linear relationship between median frequency and muscle fiber velocity. However they cannot explain the behavior of many of these variables with changes in intramuscular temperature, or muscle PH acidity, for instance. Here, we propose that the motor unit action potential can be treated as an electromagnetic resonant mode confined at thermal equilibrium inside the muscle. The motor units comprising the muscle form a system of standing waves or modes, where the energy of each mode is proportional to its frequency. Therefore, the power spectral density of the EMGA is well described and fit by Planck’s law and from its distribution we developed theoretical relationships that explain the behavior of known physiological variables with changes in intramuscular temperature or muscle PH acidity, for instance.

**Methods:**

EMGA of the calf muscle was recorded during posture maintenance in seven participants and during controlled isometric contractions in two participants. The power spectral density of the EMGA was then fit with the Planckian distribution. Then, we inferred nine theoretical relationships from the distribution and compared the theoretically derived values with experimentally obtained values.

**Results:**

The power spectral density of EMGA was fit by Planckian distributions and all the theoretical relationships were validated by experimental results.

**Conclusions:**

Only by considering the motor unit action potentials as electromagnetic resonant modes confined at thermal equilibrium inside the muscle suffices to predict known or new theoretical relationships for muscle physiological variables that other models have failed to do.

## Introduction

Electromyography activity is the electrical manifestation of neuromuscular activation. Muscle fibers are provided by end branches of the motor neuron axon, whose cell body is located in the anterior horn of the spinal grey matter [1]. The nerve cell body, its long axon, and its end branches constitute a motor unit. The ending of the axon on the muscle fiber defines an area known as the endplate. These endplates are usually, but not always, located near the middle of the muscle fibers. An action potential descending along the motor neuron activates almost simultaneously all the fibers of a motor unit (MU) [1]. When the postsynaptic membrane of a muscle is depolarized, the depolarization propagates in both directions from one of the fiber to the other. The movement of ions across the membrane during depolarization generates an electromagnetic field in the vicinity of the muscle fibers whose time excursion can be measured and the radiated power spectral density can be obtained.

The EMGA signal is usually measured by means of a bipolar electrode and constitutes the addition from many MUs signals. The electrical potentials produced by distinct MUs occur randomly and, as a consequence, a noise-like signal is produced. This random signal is often studied in the frequency domain by using the power spectrum density (PSD), for example, using summary statistics such as the median or mean frequency of the PSD [2, 3]. Since these variables reflect the whole state of a muscle, they are known as global variables [4]. The PSD of the motor neuron action potential (MUAP) depends, among other factors, on the location and configuration of the electrodes [5] and the muscle fiber velocity, *c*_*m*_. According to two models [6, 7], the relationship between *c*_*m*_ and PSD can be described as $P(\nu )=P_{0}(\nu /c_{m})/c_{m}^{2}$, where *p*_0_(*v*) is the PSD of the original waveform and *v* is the linear frequency. Thus, changes in muscle fiber velocity lead to compression or stretching of the PSD. For example, when muscular fatigue occurs the muscle fiber velocity decreases and consequently the EMGA power spectrum shifts in the direction of lower frequencies [4].

EMGA models may be generally divided in stochastic and MU-based models. Stochastic models relate the global variables to hidden anatomical and physiological parameters in the muscle [8]. They consider the EMGA to be an aleatory or stochastic process whose amplitude correlates with the level of muscle activation [4]. Stochastic models are considered successful depending how the stochastic process is mathematically described. For instance, the existing amplitude of EMGA signals measured with electrodes located on the skin can be considered as a Gaussian random variable, however, it is inadequate for describing the whole EMGA signal as a Gaussian random process [7]. This assumption is usually made because it significantly makes easier the modeling while maintaining reasonable results. Moreover, it is frequently assumed that the PSD of the EMGA signal can be written as a rational function of frequency. That is to say, the PSD is suppose to be a ratio of two polynomials in frequency, where only odd powers of frequency have zero coefficients [7]. This final supposition permits the modeled signal to be treat as filtered white noise or colored noise. Determination of the coefficients and order of the polynomials is done empirically from the PSD of EMGA signals [7].

MU-based models of the MUAP consider a muscle consisting of individual muscle fibers arranged in motor units and assembled in the same direction [6]. The fibers and the extracellular fluid comprise the substance in which the EMGA signals are distributed by volume conduction [6]. They also consider the fibers and the extracellular fluid as an isotropic, homogeneous and ohmic substance [6], conditions that are not always fulfilled. These models are capable of explaining the spatio-temporal distribution of single MUAPs throughout the muscle, which gives detailed information about MUs' architecture. However, motor-unit based models have not completely succeeded for obtaining data concerning firing rates and recruitment across the full span of contraction [4]. This is basically due to the increase in the number of MUAPs involved in the contraction and the resulting difficulty to treat the problem mathematically.

These models have successfully described many muscle physiological variables such as the value of the muscle fiber velocity *c*_*m*_ and the linear relationship between median frequency *v*_*med*_ and *c*_*m*_ [7, 9]. However they cannot explain the behavior of many of these parameters with changes in intramuscular temperature *T*_*M*_, muscle PH acidity, or to clearly identify the proportionality constants between *v*_*med*_ and *c*_*m*_. For example, if the intramuscular temperature decreases then *v*_*med*_ [10], the initial percentage median frequency %*Iv*_*med*_ [11], and *c*_*m*_[12] decreases linearly as well. There is also no EMGA model that describes that the PH of the extracellular fluid is the dominant factor in reducing *c*_*m*_ and *v*_*med*_ [13, 14]. Studies in rats showed that both the initial muscle fiber velocity and initial median frequency *Ic*_*m*_ and *Iv*_*med*_ decreased linearly when the PH of a nerve-muscle preparation was decreased.

Another important topic where there is a need for a theoretical model is muscle aging. Muscle aging is marked by a decrease in execution and fit changed circumstances. Progressive inability of regeneration machinery to replace damaged muscles is a sign of aging related to sarcopenia or muscle loss [15]. This characteristic is shared with other conditions that involve muscle declining, such as AIDS, amyotrophic lateral sclerosis and cancer, all distinguished by physiological and metabolic alterations [15]. The metabolism of a person is the result of all chemical reactions that the human body can make and spend. The rate of decrease of the internal energy in the body is known as metabolic rate. The basal metabolic rate (*BMR*) is defined similarly to the metabolic rate but it is measured in the morning when a subject is awake, at ambient temperature, lying down in a bed and without any meals ingested. The resting skeletal muscles account for approximately one fifth of the *BMR* [16]. Moreover, the *BMR* per surface area (*BMRS*) decreases as we age [17]. An intriguing experimental result is presented in a recent aging study [18] where neuromuscular performance in young and aged subjects was studied by tracking the changes of the EMGA PSD *v*_*med*_ and *c*_*m*_ while the subjects performed maximal voluntary contractions (MVC). Both *v*_*med*_ and *c*_*m*_ in aged subjects decreased when compared with young subjects. Clearly a decreasing metabolism due to aging may decrease *T*_*M*_, having as a consequence the decrease in *v*_*med*_ and *c*_*m*_. A new EMGA PSD theoretical model should include these metabolic effects. Currently there is no model that explains the shift in *v*_*med*_ and *c*_*m*_ with aging.

What is more, in 1912, Piper [1] discovered that the PSD of the EMGA compresses and shifts towards low frequencies during a sustained contraction. Present-day models predict both the compression in the EMGA PSD during sustained contractions, and the shift in the spectrum towards lower frequencies [6, 7]. Therefore any new EMGA PSD model should also describe muscular fatigue if it is going to be considered as a good model. So clearly, there is a need to improve the current EMGA PSD theoretical models to explain the aforementioned experimental relationships and also describe what it is already known such as muscle fiber fatigue.

Planck’s energy radiation law reports the spectral distribution of energy in a cavity that wholly absorbs all radiant energy impinging upon it, reaches thermal equilibrium, and then reemits that energy as quickly as it absorbs it (blackbody or cavity radiation). The radiated energy can be considered to be the product of standing waves or resonant modes of the radiating cavity. The occurrence of standing waves in muscular fiber is well known. From the pioneering work of Eben in 1936 [19], it has been shown that the spiking rate of motor nerve endplates regulates the arrangement of emanated mechanical pressure waves through the muscle fibers, conveyed as cross-striations, and then these waves are reflected at both fibers’ ends. The interaction of incident and reflected waves form a complex stationary wave system in both the transversal and perpendicular directions of the fiber. Therefore, the energy state of a muscle can be characterized by specifying the different possible types of standing waves. As the energy increases, the frequency of muscle fiber oscillations increases and the wavelengths become shorter. When the energy decreases, there is a corresponding decrease of wave frequency accompanied by longer wavelengths. The standing waves in muscle fibers can be related to resonant modes in a radiative cavity through Planck's Law. Therefore we will consider that EMGA power spectral densities may obey the Planckian distribution. Also, Planck’s radiation law does not depend on any random process or variable, and it is not the result of any ratio of two arbitrary polynomials in frequency, where the order and coefficients of the polynomials are determined empirically. It does not require the assumption that muscle fibers are organized in one direction or that the medium where the EMGA signals are conducted be homogeneous, isotropic, homogeneous, and ohmic. It only requires thermal equilibrium of resonant modes within the cavity. So, it does not matter how complex the cavity is or what it is made of. These properties would help explore different levels of isometric contractions in muscle fibers by just tracking distribution changes with each contraction.

For isometric contractions the Planckian distribution can be obtained then by considering that the MUAPs behave as electromagnetic resonant modes confined at thermal equilibrium in a muscle temperature range from 10 up to 37°C [11], where they form a system of standing waves and where the energy of each mode is proportional to the standing wave frequency. The theoretical formula (see S1 File) for the radiated power through a surface of certain area at some temperature is given by the Planckian distribution

$$P(\nu ,dT)=\frac{\pi Sh}{c_{m}^{2}}\left(\frac{\nu^{3}}{e^{\frac{\nu }{adT}}-1}\right)=\frac{\pi Sh}{{\left(2ladT\right)}^{2}}\left(\frac{\nu^{3}}{e^{\frac{\nu }{adT}}-1}\right),$$ (1)

where *S* is the area of the emission surface in *m* ^2^,*h* is the equivalent of Planck's constant in *V* ^2^/*Hz*, *c*_*m*_ is the muscle fiber velocity in *m*/*s*, given by 2*ladT*, *a* is a constant in *Hz*/°*C* or *Hz*/*K*,*v* is the frequency in *Hz*, the parameter *l* represents the average fiber length. Notice that Eq 1 is inversely proportional to $c_{m}^{2}$ as in the models presented in [6, 7]. The term *adT* represents the muscle characteristic frequency *v*_0_. When this characteristic frequency is multiplied by constant *h*, the product *hadT* is proportional to the muscle thermal energy *E*_*TH*_, where *dT* is the change between *T*_*M*_ in °*C* or *K* and the intramuscular absolute initial temperature *T*_M0_ in °*C* or *K*, i.e. *dT* = (*T*_*M*_-*T*_M0_). The value for *T*_M0_ can be taken as 0°*C* or 273 *K* (see S1 File). Given that most of the physiological experiments that relate to temperature use degrees Celsius, in the following sections we will use only degrees Celsius instead of Kelvin.

The present work is divided in six phases: first, we show that the EMGA power spectral density in the Gastrocnemius medial muscle is fitted very well by a Planckian distribution during posture maintenance and controlled isometric contractions. The distribution requires only a few parameters (*S*,*h*, *a*, *dT*) whose meaning is relatively straightforward. Second, we experimentally obtained *c*_*m*_ by hypothesizing the existence of standing waves or modes and then compared its value with known results presented in [2, 7]. We also showed the relationship between *c*_*m*_ vs. *dT* and compared it with data found in [12]. Third, we experimentally obtained *v*_*max*_, *v*_*med*_, %*Iv*_*med*_, and the irradiance *I* then performed multiple regression analyses to test which parameters *S*,*h*, *a*, *dT* were significant predictors of *v*_max_, *v*_*med*_, %*Iv*_*med*_, and *I*. We found *dT* is the best predictor and performed a linear fit between *v*_max_, *v*_*med*_,%*Iv*_*med*_ vs. *dT* or a power fit for *I* vs. *dT*. We also performed multiple regression analyses to test which parameters *S*,*h*, *a*, *c*_*m*_ were significant predictors of *v*_*med*_. We found *c*_*m*_ is the best predictor and generated a linear fit between *v*_*med*_ vs. *c*_*m*_. Fourth, from the Planckian distribution we developed theoretical relationships between *v*_max_, *v*_*med*_, %*Iv*_*med*_ and *I* vs. *dT*, *v*_*med*_ vs. *c*_*m*_, *Iv*_*med*_ and *Ic*_*m*_ vs. muscle PH, *v*_*med*_, *c*_*m*_ vs. human aging, and finally *v*_*med*_, *c*_*m*_ and PSD amplitude at *v*_*med*_ versus muscle fatigue. Fifth, we compared the regression models against the theoretical relationship for *v*_max_, *v*_*med*_, %*Iv*_*med*_, and *I* vs. *dT*, and *v*_*med*_ vs. *c*_*m*_. The relationship *v*_*med*_ vs. *dT* was also compared with the experimental relationship found in [10]. The relationship %*Iv*_*med*_ vs. *dT* was also compared with data extracted from [11]. The relationship *v*_*med*_ vs. *c*_*m*_ was also compared with data found in [9]. Sixth, we used the Planckian distribution to develop theoretical relationships for *Iv*_*med*_ and *Ic*_*m*_ vs. muscle PH, *v*_*med*_, *c*_*m*_ vs. human aging and *v*_*med*_, *c*_*m*_ and PSD amplitude at *v*_*med*_ vs. muscle fatigue and compared these theoretical results against experimental studies found in [14],[18] and [1, 13] respectively. If the agreement is good in all these comparisons then a Planckian distribution not just fits EMGA power spectral densities well in muscles but it will extend the current knowledge we have on isometric contractions because this distribution predicts new theoretical relationships that other models have failed to do. Moreover, previous known theoretical relationships obtained from models other than a Planckian distribution are also well described by a Planckian distribution.

## Materials and Methods

### General

An EMGA electrode was inserted into the right Gastrocnemius medial muscle of N = 9 participants. The electrode was placed far from the innervation zones which are located either at the perimeter of the muscle or at one end of the muscle [13, 20]. In the first condition (Sharpened Romberg Position), participants were required maintain balance while standing in a tandem heel-to-toe position with eyes open and with arms folded against the chest [21]. This position is inherently unstable and allowed us to examine different contraction ranges of the muscle. In the second condition we asked N = 2 participants to execute isometric contractions of their right gastrocnemius medial at 10% of the maximum voluntary contraction (MVC) while sitting comfortably with eyes open. This allowed us to have better control on the contraction range of a muscle.

The Bagnoli (DELSyS, Inc.) handheld EMGA tracking system was used to measure EMGA.

For the Sharpened Romberg Position task, EMGA was tracked for 10 trials each lasting 35 seconds (one-minute pause between trials) and for the maximum voluntary contraction task, EMGA was tracked for 10 trials, each consisting of 10 contractions of 1 second with one-minute pauses between trials.

The study obtained ethics approval from the CERES (Comité d’éthique de la recherche en santé) of Université de Montréal, where all the testing took place. Informed written consent was obtained from all participants of the study.

### Planckian distribution fittings (I)

The PSD was calculated by means of a FFT algorithm for each trial and then averaged for each subject. All the averaged PSDs were then fitted with parameters *S*,*h*,*a*, and *dT* from Eq 1. The parameter *l* that represents the average gastrocnemius medial fiber length was taken from reference [22]. For the fitting we used the non-linear least squares method implemented in Matlab's curve-fitting tool. We use a parametric nonlinear regression model of Eq 1, the dependent variable or the response is the EMGA PSD, the independent variable is the linear frequency *v* (predictor) and the non-linear parameters were *S*,*h*,*a*, and *dT*. To determine the nonlinear parameter estimates, we used the function *y*_*i*_ = P(*v*_*i*_,*S*,*h*,*a*,*dT*)+*ε*_*i*_, where *y*_*i*_,*v*_*i*_ and *ε*_*i*_ represent the *i*-*th* numerical PSD value, frequency and residual or error. The function that is minimized is given by $\sum_{i=1}^{m}\varepsilon {\left(x\right)}_{i}{}^{2}=\sum_{i=1}^{m}{\left(y_{i}-P(\nu_{i},S,h,a,dT)\right)}^{2}$, where *x* is a vector given by *x* = (*S*,*h*,*a*,*dT*). Nonlinear models are more difficult to fit than linear models because the coefficients cannot be estimated using simple matrix techniques. Instead, an iterative approach is required as follows: Start with an initial estimate for each coefficient. Produce the fit for the current set of coefficients. Adjust the coefficients and determine whether the fit improves. The direction and magnitude of the adjustment depend on the fitting algorithm. Here, we have used the Trust-region algorithm [23, 24] (see S1 File).

### *c*_*m*_ values and theoretical relationship between *c*_*m*_ vs. *dT* (II)

*c*_*m*_ was obtained experimentally from the EMGA PSD for each subject. *c*_*m*_ was estimated by working in the frequency region where *v*≤*adT*. There, we can consider that all the MUAP frequencies fluctuate around an average value of *v*_0_ = *adT*. We can then assume that *v*_0_ corresponds to the frequency of a standing wave with wavelength *λ* = 2*l* where *l* is the average muscle fiber length. From this assumption, we can calculate the muscle fiber velocity as *c*_*m*_ = 2*ladT*. The parameter *l* that represents the average gastrocnemius medial fiber length was taken from reference [22]. Clearly the expression *c*_*m*_ = 2*ladT* represents a linear relationship between *c*_*m*_ and *dT* with a theoretical sensitivity or slope of 2*la* in *m*/*s*°*C*.

### Regression Models (III)

#### *v*_max_ vs. *S*,*h*, *a*, *dT*

*v*_max_ was obtained experimentally from the EMGA PSD for each subject. At this frequency the EMGA PSD reaches its maximum. Then we performed multiple regression analyses using SPSS to test which parameters *S*,*h*, *a*, *dT* of the Planckian distribution are significant predictors of *v*_max_. Pearson correlations and ANOVA methods were used to determine the best predictor (*dT*). Once found, we performed a linear fit between *v*_max_ and best predictor.

#### *v*_med_ vs. *S*,*h*, *a*, *dT*

*v*_med_ was obtained experimentally from the EMGA PSD for each subject. At this frequency the EMGA PSD area under the curve is half (see S1 File). Then we performed multiple regression analyses using SPSS to test which parameters *S*,*h*, *a*, *dT* of the Planckian distribution are significant predictors of *v*_med_. Pearson correlations and ANOVA methods were used to determine the best predictor (*dT*). Once found, we performed a linear fit between *v*_*med*_ and best predictor.

#### %*Iv*_*med*_ vs. *S*,*h*, *a*, *dT*

*v*_med_ was obtained experimentally as explained above. Without loss of generality we can choose any value for *v*_*med*_ as reference value *v*_*medI*_ from the EMGA PSD for each subject, here we choose the highest value found in Table 1 (154 Hz). Then we divide each *v*_*med*_ value with *v*_*medI*_ and multiply the result by 100 to obtain the initial percentage median frequency %*Iv*_*med*_. Then we performed multiple regression analyses using SPSS to test which parameters *S*,*h*, *a*, *dT* of the Planckian distribution are significant predictors of %*Iv*_med_. Pearson correlations and ANOVA methods were used to determine the best predictor (*dT*). Once found we performed a linear fit between %*Iv*_*med*_ and best predictor.

**Table 1:** Fitting parameters values and dependent variables maximum frequency *v*_max_, median frequency *v*_*med*_ and irradiance *I*.

		Best fit parameters					*R* ^2^	Dependent variables		
		*h*	*S*	*c*_*m*_	*a*	*dT*		*I*	*v*_max_	*v*_*med*_
Subjects	S1	1.59E-13	4.89E-4	4.158	1.187	27.80	0.9605	2.3E-7	93.62	119.00
	S2	1.59E-13	1.15E-4	3.402	1.186	22.70	0.9354	1.5E-7	75.00	093.00
	S3	1.59E-13	1.42E-4	3.276	1.189	21.86	0.9244	1.7E-7	65.00	106.00
	S4	1.59E-13	2.89E-4	2.646	1.191	17.63	0.9409	1.1E-7	54.18	091.00
	S5	1.59E-13	1.00E-4	3.780	1.188	25.25	0.9531	2.1E-7	86.33	122.00
	S6	1.59E-13	1.92E-4	5.544	1.190	36.97	0.9872	3.8E-7	127.99	153.00
	S7	1.59E-13	1.29E-4	3.654	1.192	24.33	0.9399	2.0E-7	83.00	116.00
	S8	1.59E-13	5.39E-4	5.166	1.189	34.48	0.9833	3.4E-7	110.00	146.00
	S9	1.59E-13	5.48E-4	5.544	1.190	36.97	0.9735	3.9E-7	103.00	154.00
Average		1.59E-13	2.83E-4	4.130	1.189	27.55		2.4E-7	88.68	122.22
Error		0	6.36E-5	0.351	0.001	02.34		3.4E-8	07.64	008.05

Show the values for best-fit parameters *h*, *S*, *c*_*m*_, *a*, and *dT*, as well as the R-square the model fit for each subject. The last three columns show the model’s dependent variables obtained from the PSD distribution: the irradiance *I*, maximum frequency *v*_max_, and median frequency *v*_*med*_. The goodness-of-fit of the model was very high, as reflected in the high R-square values. Furthermore, the value of parameters *h* and *a* were virtually identical across participants, indicating that these parameters can be considered as constants. The error is the standard error.

#### *v*_med_ vs. *S*,*h*, *a*, *c*_*m*_

*v*_*med*_ and *c*_*m*_ were obtained experimentally as described above. Then we performed multiple regression analyses using SPSS to test which parameters *S*,*h*, *a*, *c*_*m*_ of the Planckian distribution are significant predictors of *v*_*med*_. Pearson correlations and ANOVA methods were used to determine the best predictor (*c*_*m*_). Once found we performed a linear fit between *v*_*med*_ and best predictor.

#### *I* vs. *S*,*h*, *a*, *dT*

*I* was obtained experimentally from the EMGA PSD for each subject. To obtain the irradiance *I*, we performed a numerical integration on every subject's average PSD and divided the result by its corresponding emission surface area *S*. Multiple linear regression analysis was used to develop a model for predicting the logarithm of irradiance from the logarithm of parameters *S*,*h*, *a*, *dT*. Pearson correlations and ANOVA methods were used to determine the best predictor (*dT*). Once found we performed a power fit between *I* and best predictor.

### Theoretical relationships inferred from Planck’s distribution (IV)

#### *v*_max_ vs. *dT*

The Planckian distribution has its maximum at a frequency determined by *v*_max_ = 2.821*adT* (see S1 File), which represents a linear relationship with a sensitivity or slope of 2.821*a Hz*/°*C*.

#### *v*_*med*_ vs. *dT*

The Planckian distribution has its median at a frequency given by *v*_*med*_ = 3.503*adT* (see S1 File), which represents a linear relationship with sensitivity or slope of 3.503*a Hz*/°*C*.

#### %*Iv*_*med*_ vs. *dT*

Theoretically, %*Iv*_*med*_ is given by 100*dT*/(*T*_*MI*_−*T*_M0_) (see S1 File), where *T*_*MI*_ is the initial reference temperature and the sensitivity (slope) is then given by 100/(*T*_*MI*_−*T*_M0_).

#### *v*_*med*_ vs. *c*_m_

We can substitute the relationship *c*_*m*_ = 2*ladT* into the relationship *v*_*med*_ = 3.503*adT* to obtain, *v*_*med*_ = 3.503*c*_*m*_/2*l*, thus revealing that the median frequency *v*_*med*_ depends linearly on the muscle fiber velocity *c*_*m*_ with a slope of 3.503/2*l* in *Hz*/*m*/*s*.

#### *I* vs. *dT*

The irradiance is obtained by integrating the Planck distribution across the range of all possible frequencies and then divide the result by the surface of area *S* (see S1 File). The final result is

$$I\approx \frac{h\pi^{5}a^{2}dT^{2}}{60l^{2}}$$ (2)

#### *Iv*_*med*_ and *Ic*_*m*_ vs. muscle PH

Now, by assuming the parameter*a* is not constant anymore but a PH function, we can write *v*_*med*_ = 3.503*a*(*PH*)*dT* and *c*_*m*_ = 2*la*(*PH*)*dT*. From these two expressions, we obtain that the initial median frequency and the initial muscle fiber velocity depends on the muscle *PH* as, *Iv*_*med*_ = *Ic*_*m*_ = *a*(*PH*)/*a*(*PH*_*I*_) where *PH*_*I*_ represents the initial *PH* condition (see S1 File). If we assume a simple linear approximation for *a*(*PH*) equal to *αPH*, where *α* is a constant, we should expect that *Iv*_*med*_ = *Ic*_*m*_ = *PH/PH*_*I*_, which represent two linear relationships with identical slopes given by 1/*PH*_*I*_.

#### *v*_*med*_ and *c*_*m*_ vs. muscle aging

If we assume that the thermal energy *E*_*TH*_ is proportional to the *BMR* (see S1 File), so any relative change *δE*_*TH*_/*E*_*TH*_ of this energy should be equal to *δBMR*/*BMR* and because *E*_*TH*_, *c*_*m*_ and *v*_*med*_ are proportional to *dT*, therefore *δBMR*/*BMR* = *δv*_*med*_/*v*_*med*_ = *δc*_*m*_/*c*_*m*_. Since *BMR* = (*BMRS*)*BSA*, where *BSA* is the human body surface area, therefore *δBMR*/*BMR* = *δBMRS*/*BMRS*+*δBSA*/*BSA*, so the relative change or BMR due to aging is given by the BMRS relative change plus the BSA relative change (related to sarcopenia or muscle loss). By knowing these relative changes with age we can know the relative changes with age of *v*_*med*_ and *c*_*m*_. That is, any relative change on the basal metabolic rate due to aging should be reflected exactly in the same proportion in *v*_*med*_ and *c*_*m*_.

#### *v*_*med*_, *c*_*m*_ and the PSD amplitude evaluated at *v*_*med*_ vs. muscle fatigue

The Planckian distribution can predict the fatigue effects on muscle fibers by considering 1) that the EMGA PSD median frequency and the muscle fiber velocity values decrease with muscle fatigue and 2) that the compression of the EMGA PSD results in an increase of the PSD amplitude evaluated at the median frequency. Mathematically (see S1 File), the changes *δc*_*m*_ of the muscle fiber velocity, *δv*_*med*_ of the median frequency, and *δP*_*med*_ of the Planckian distribution amplitude evaluated at the median frequency can be used to describe fatigue effects in muscle fibers when*δP*_*med*_≤0, *δc*_*m*_≤0 and *δP*_*med*_≥0. This last inequality gives 3|*δv*_*med*_/*v*_*med*_|≤−2*δc*_*m*_/*c*_*m*_, where the brackets denote the absolute value.

## Results

### Planckian distribution fits (I)

**Fig 1:**
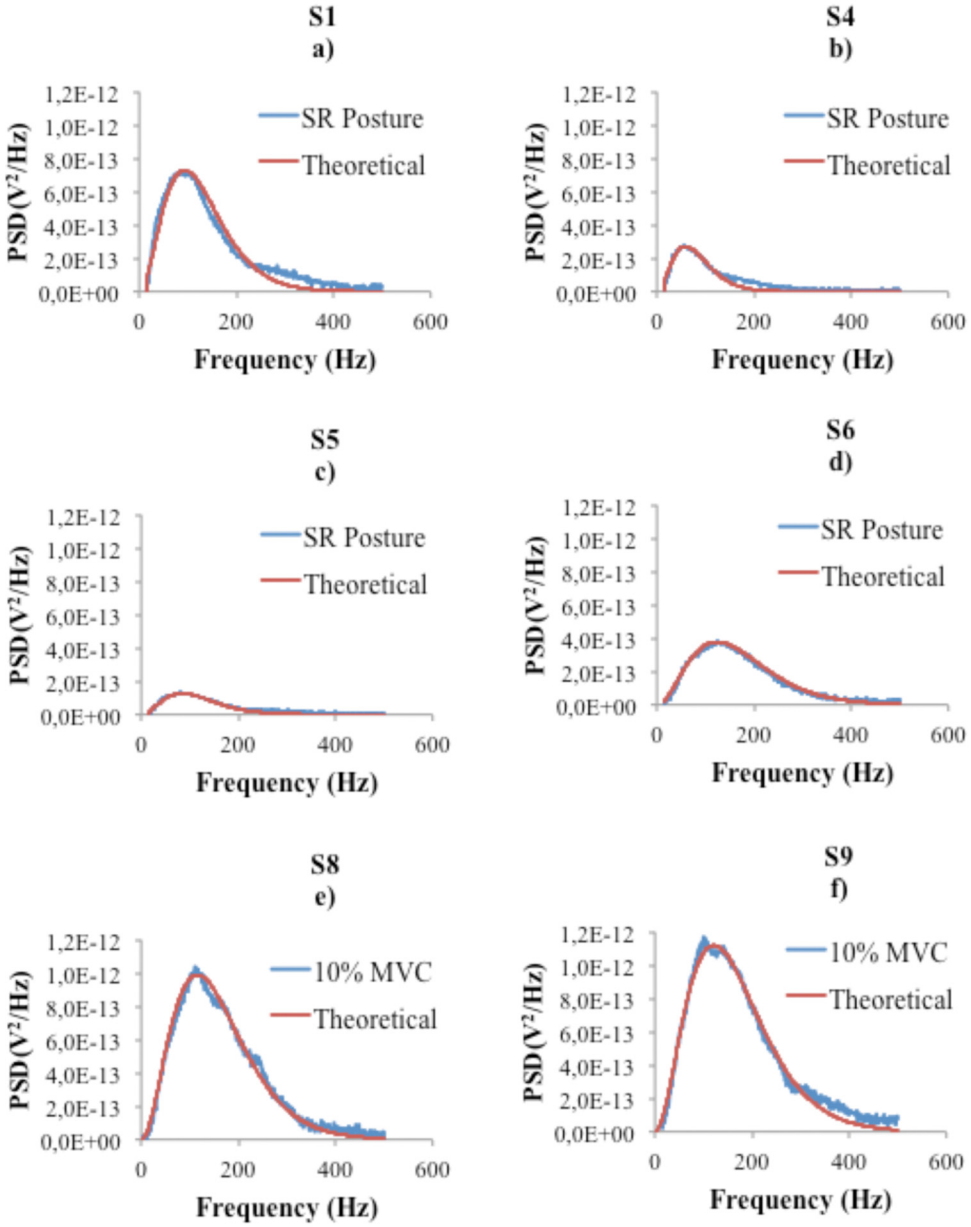
Comparison between experimental and theoretical Power Spectrum Densities in four subjects for the posture maintenance task (first and second row) and two subjects for the 10% MVC task (third row).

Fig 1 shows the fit results for the SR position task (four participants) and the MVC task (two participants) respectively. All parameters and the R-squared coefficients are shown in Table 1. We can observe that the fits are very good, a fact that is corroborated by the high R-squared coefficients' values. Moreover, it can be seen that the best-fit values for *h* and *a* are very similar across participants, indicating that these parameters are in fact constants.

In order to show that this distribution is capable of reproducing known electrophysiological results or predict new ones, we now take all the theoretical relationships inferred from the Planckian distribution and compare them against either the fit obtained with multiple regression analysis or experimental results obtained elsewhere. A summary of these results is shown in Table 2.

**Table 2:** Comparison between theoretical relationships inferred from Planck’s distribution and experimental physiological results obtained from our data or someone else’s.

Function	Theoretical relationship	Data from this work	Other groups
*v*_max_(*dT*)	*v*_max_ = 2.821*adT* = 3.36*dT*	^1^ *v*_max_ = (3.21±0.09)*dT*	
*v*_*med*_(*dT*)	*v*_max_ = 3.503*adT* = 4.17*dT*	^1^ *v*_med_ = (4.37±0.11)*dT*	^2^ *v*_*med*_ = (4.05±0.16)*dT*
			^2^ *v*_*med*_ = (3.55±0.05)*dT*
			^2^ *v*_*med*_ = (3.23±0.25)*dT*
			^2^ *v*_*med*_ = (3.93±0.24)*dT*
			^2^ *v*_*med*_ = (4.07±0.29)*dT*
%*Iv*_*med*_(*dT*)	^3^%*Iv*_*med*_ = [100/(*T*_*MI*_−*T*_M0_)]*dT* = 2.71*dT*	^1^%*Iv*_*med*_ = (2.84±0.07)*dT*	
	^4^%*Iv*_*med*_ = [100/(*T*_*MI*_−*T*_M0_)]*dT* = 3.03*dT*		^5^%*Iv*_*med*_ = 3.03*dT*
*c*_*m*_(*dT*)	^6^ *c*_*m*_ = 2*ladT* = (0.23±0.02)*dT*		^7^ *c*_*m*_ = 0.2*dT*
$\bar{c_{m}}$	$\bar{c_{m}}={\langle c_{m}\rangle }_{dT}={\langle 2ladT\rangle }_{dT}$	^1^ ^$\bar{c_{m}}=(4.13\pm 0.35)m/s$^	^8^ ^$\bar{c_{m}}=(4)m/s$^
*v*_*med*_(*c*_*m*_)	^1^ *v*_*med*_ = (3.503/2*l*)*c*_*m*_ = 27.8*c*_*m*_	^1^ *v*_*med*_ = (29.19±0.75)*c*_*m*_	
	^9^ *v*_*med*_ = (3.503/2*l*)*c*_*m*_ = 25.8*c*_*m*_		^10^ *v*_*med*_ = 23.1*c*_*m*_
*I*(*dT*)	^1^ *I*≈*hπ* ^5^ *a* ^2^ *dT* ^2^/60*l* ^2^ = (2.88×10^-10^x1.10×10^−10^)*dT* ^2^	^1^ *I* = (8.99×10^−10^±2.69×10^−10^)*dT* ^1.7±0.1^	
*Iv*_*med*_(*Ic*_*m*_)	*Iv*_*med*_ = *Ic*_*m*_		^11^ *Iv*_*med*_ = 1.07*Ic*_*m*_
*Iv*_*med*_(*PH*)	^12^ *Iv*_*med*_ = (1/*PH*_*I*_)*PH* = 0.135*PH*		^11^ *Iv*_*med*_ = 0.134*PH*
*Ic*_*m*_(*PH*)	^12^ *Ic*_*m*_ = (1/*PH*_*I*_)*PH* = 0.135*PH*		^11^ *Ic*_*m*_ = 0.134*PH*
*v*_*med*_(*Age*)	*δBMR*/*BMR* = *δBMRS*/*BMRS*+*δBSA*/*BSA*		^14^ *δv*_*med*_/*v*_*med*_ = 0.18±0.03
	^13^ *δBMRS*/*BMRS* = 0.13		
	^1^ ^,^ ^13^ *δBSA*/*BSA* = 0.2		
	*δBMR*/*BMR* = 0.13+0.2 = 0.15		
	*δv*_*med*_/*v*_*med*_ = *δBMR*/*BMR* = 0.15		
*c*_*m*_(*Age*)	*δBMR*/*BMR* = *δBMRS*/*BMRS*+*δBSA*/*BSA*		^14^ *δc*_*m*_/*c*_*m*_ = 0.19±0.07
	^13^ *δBMRS*/*BMRS* = 0.13		
	^1^ ^,^ ^13^ *δBSA*/*BSA* = 0.2		
	*δBMR*/*BMR* = 0.13+0.2 = 0.15		
	*δc*_*m*_/*c*_*m*_ *= δBMR*/*BMR* = 0.15		
**Fatigue**	*δv*_*med*_≤0	15	16
	*δv*_*med*_≤0		
	^3^|*δv*_*med*_/*v*_*med*_|≤−2*δc*_*m*_/*c*_*m*_		

Constant values used for theoretical calculations *α* = 1.1891,*h* = 1.59×10^−13^,*T*_M0_ = 0°*C*.

^1^ *l* taken from reference [22].

^2^Reference [10], 20%,40%,60%, 80% and 100% MVC.

^3^T_MI_ = 36.9°C taken from Table 1.

^4^T_MI_ = 33°C taken from Reference [11].

^5^Reference [11].

^6^ *l* taken from reference [25].

^7^Reference [12].

^8^References [1, 2].

^9^ *l* taken from reference [26].

^10^Reference [9].

^11^Reference [14].

^12^ *PH*_*I*_ taken from reference [14].

^13^Reference [17].

^14^Reference [18].

^15^ Fig 8.

^16^References [1, 13].

### Comparison of *c*_*m*_ values obtained here vs. other studies and comparison between the theoretical relationship between *c*_*m*_ vs. *dT* and other studies (II)

Experimentally, *c*_*m*_ values ranges from 2 to 6 m/s [2, 7] and average value of 4 m/s [1, 2]. In Table 1 we observe the values we obtained for *c*_*m*_. The values ranged from 3.28 to 5.54 m/s with average of 4.13±0.35 m/s, which is close to the average value that is normally used. Working with the human Vastus medialis muscle, Morimoto and colleagues [12] found a linear relationship between the muscle fiber velocity and the intramuscular temperature, with a sensitivity of 0.2 *m*/*s*°*C* in the range of 17–31°C. By using a length for the human Vastus medialis fibers [25] of 9.53±0.63 cm and the average value of 1.1891 for *a* (see Table 1) we found a theoretical value for the slope 2*la* of 0.23±0.02 *m*/*s*°*C*, which is close to the experimental values obtained by Morimoto and colleagues.

### Comparison between regression models vs. theoretical relationships inferred from Planck’s distribution (V)

#### *v*_max_ vs. *dT*

**Fig 2:**
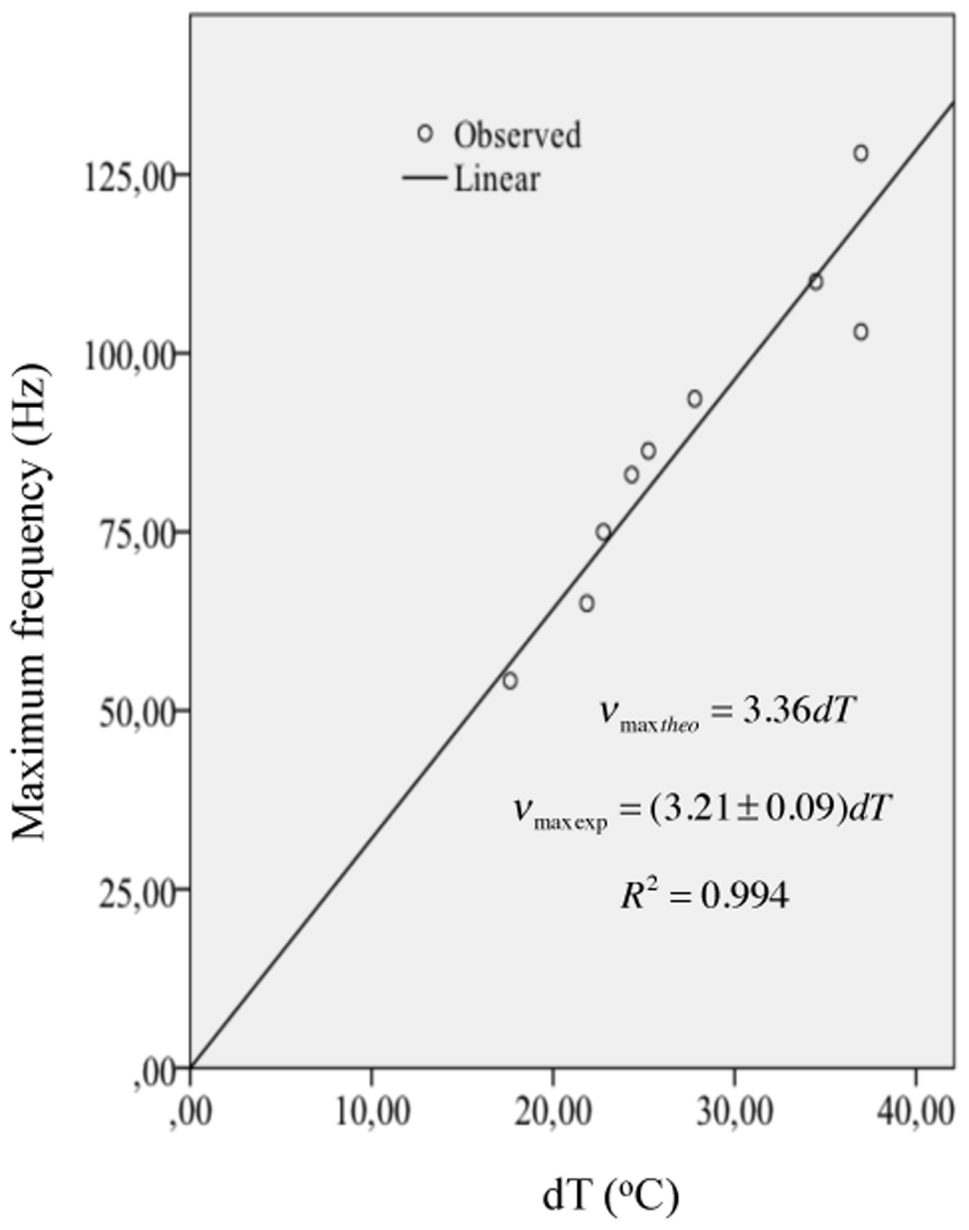
Relationship between maximum frequency and parameter *dT*. The slope value of 3.21±0.09 *Hz*/°*C* represents a difference of less than 5% with respect to the theoretical value of 3.36 *Hz*/°*C*.

To evaluate the relationship between model parameters *S*, *h*, *a*, *dT* and *v*_max_, we conducted multiple linear regression analysis with *v*_max_ as the dependent variable and parameters *S*, *h*, *a*, and *dT* as predictors. Table 3 shows the results of these analyses. Only *dT* had a significant (*p* < 0.01) Pearson correlation with *v*_max_ and only *dT* was found to be a significant (*p* <0.01) predictor in the full linear regression model. The predictor model was able to account for 92.7% of the variance in *v*_max_, *F(1*,*8)* = 21.27, *p* = 0.003, *R* ^*2*^ = 0.927. Thus, *v*_max_ depends largely on *dT*. We performed a linear fit between *v*_max_ and *dT* and found a slope value of 3.21±0.09 *Hz*/°*C*. This result supports the theoretical equation *v*_*max*_ = 2.821*adT*. If we use the average value of 1.1891 for *a* (see Table 1) we obtain a slope value of 3.36 *Hz*/°*C* which is of the same order of magnitude as 3.21±0.09 *Hz*/°*C*. The experimental data and the linear fit are shown in Fig 2.

**Table 3:** Person correlation and beta values for predicting the maximum frequency *v*_max_ from parameters*S*,*h*,*a*, and *dT*.

Person correlation coefficients						Linear model
	Fit parameters			Fit parameters	*v*_max_	*β* values
Fit parameters	*dT*	*a*	*S*			
*S*				*S*	0.383	-0.203
*a*			0.001	*a*	0.003	-0.510
*dT*		0.051	0.552	*dT*	0.947*	1.062**

Shows Person correlation and beta values obtained from multiple linear regression analysis Only *dT* had a significant (*p < 0.01) Pearson correlation with *v*_max_ and only *dT* was found to be a significant (**p <0.01) predictor in the full linear regression model. The predictor model was able to account for 92.7% of the variance in *v*_max_, *F(1*,*8)* = 21.27, *p* = 0.003, *R* ^*2*^ = 0.927.

#### *v*_*med*_ vs.*dT*

**Fig 3:**
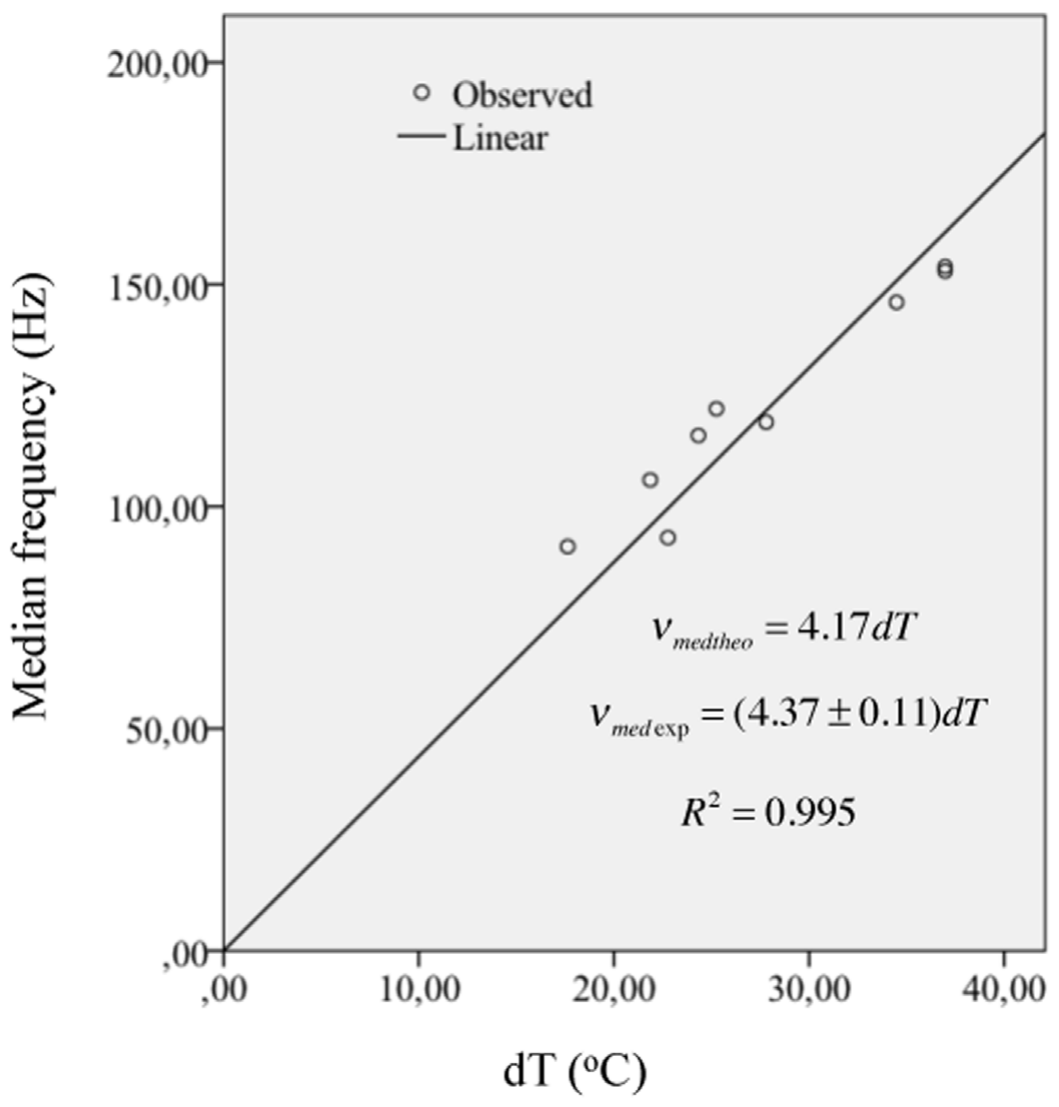
Relationship between medium frequency and parameter *dT*. The slope value of 4.37±0.11 *Hz*/°*C* represents a difference of less than 5% with respect to the theoretical value of 4.17 *Hz*/°*C*.

Multiple linear regression analysis was used to develop a model for predicting *v*_*med*_ from parameters *S*, *h*, *a*, and *dT*. Table 4 shows the results of these analyses. Only *dT* had a significant (*p* < .01) Pearson correlation with *S*, *h*, *a*, and *dT* and only *dT* predictor had a significant effect (*p* <0.01) in the full model. The predictor model was able to account for 96.5% of the variance in *v*_*med*_, *F(3*,*8)* = 45.63, *p*<0.001, *R* ^*2*^ = 0.965. As a consequence of this result, we performed a linear fit between *v*_*med*_ and *dT* only and obtained a slope of 4.37±0.11 *Hz*/°*C*. This result supports the theoretical equation *v*_*med*_ = 3.503*adT*. If we use the average value of 1.1891 for *a* (see Table 1) we obtain a slope value of 4.17*Hz*/°*C* that is of the same order of magnitude as 4.37±0.11 *Hz*/°*C*. The experimental data and the linear fit are shown in Fig 3. In a recent study [10] Petrofsky and Laymon studied the effect of temperature on the EMGA PSD amplitude. The EMGA was measured over several muscles, including the one we have studied here (Gastrocnemius medial). Short (3s) isometric contractions were executed at different tensions ranging between 20 and 100% of each subject’s maximum strength. Placing the arm or leg of the subjects in water. The water bath temperature was controlled at 24, 27, 34, and 37°C during those contractions. The results showed that the median frequency of the EMGA PSD was directly proportional to the temperature of the Gastrocnemius medial muscle amid the succinct isometric contractions [10] with sensitivities of 4.05±0.16 Hz/°C, 3.55±0.05 Hz/°C, 3.23±0.25 Hz/°C, 3.93±0.24 Hz/°C, 4.07±0.29 Hz/°C, for 20, 40, 60, 80 and 100% MVC. All these experimental values are of the same order of magnitude as the theoretical value of 4.17 Hz/°C.

**Table 4:** Person correlation and beta values for predicting the median frequency *v*_*med*_ from parameters*S*,*h*,*a*, and *dT*.

Person correlation coefficients						Linear model
	Fit parameters			Fit parameters	*v*_med_	*β* values
Fit parameters	*dT*	*a*	*S*			
*S*				*S*	0.517	-0.021
*a*			0.001	*a*	0.206	-0.157
*dT*		0.051	0.552	*dT*	0.969*	0.973**

Shows Person correlation and beta values obtained from multiple linear regression analysis. Only *dT* had a significant (*p < .01) Person correlation with *v*_*med*_ and only *dT* predictor had significant (**p <0.01) effect in the full model. The predictor model was able to account for 96.5% of the variance in *v*_*med*_ *F(3*,*8)* = 45.63, *p*<0.001, *R* ^*2*^ = 0.965.

#### %*Iv*_*med*_ vs. *dT*

**Fig 4:**
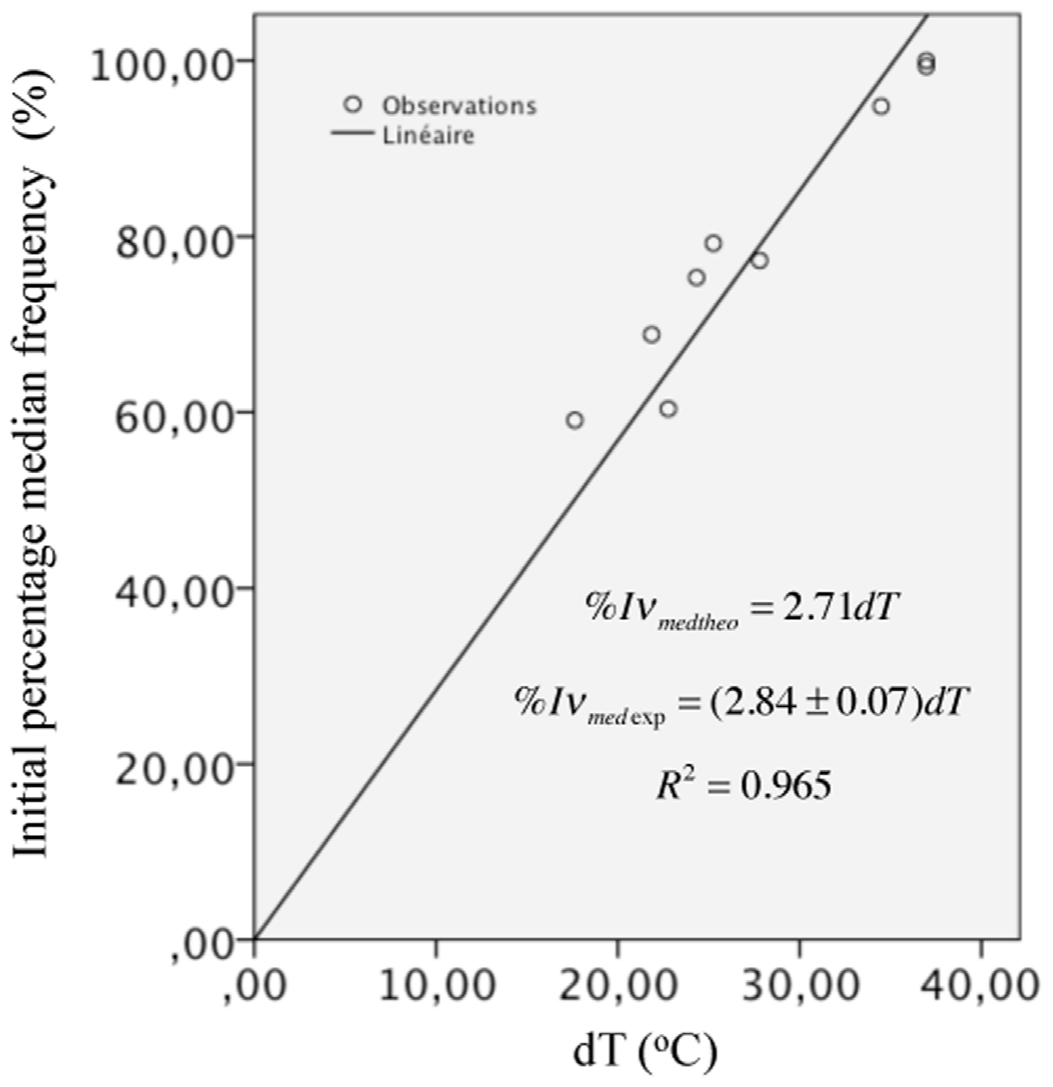
Relationship between the initial percentage median frequency %*Iv*_*med*_ and parameter *dT*. The slope value of 2.84±0.07%/°*C* represents a difference of less than 5% with respect to the theoretical value of 2.71%/°*C*.

Multiple linear regression analysis was used to develop a model for predicting %*Iv*_*med*_ from parameters *S*, *h*, *a*, and *dT*. Table 5 shows the results of these analyses. Only *dT* had a significant (*p* < .01) Pearson correlation with %*Iv*_*med*_ and only *dT* predictor had a significant effect (*p* <0.01) in the full model. The predictor model was able to account for 96.5% of the variance in %*Iv*_*med*_, *F(3*,*8)* = 45.63, *p*<0.001, *R* ^*2*^ = 0.965. As a consequence of this result, we performed a linear fit between %*Iv*_*med*_ and *dT* only and obtained a slope of 2.84±0.07%/°*C*. This result supports the theoretical equation 100*dT*/(*T*_*MI*_-*T*_M0_). If we use the value for *dT*_*MI*_ associated with *v*_*medI*_ = 154Hz that equals to36.97°C (see Table 1) we obtain a slope value of 100/(36.97−0) = 2.71%/°*C* that is of the same order of magnitude as 2.84±0.07%/°*C*. The experimental data and the linear fit are shown in Fig 4. We also used the data reported by Merletti and colleagues [11] from experiments where they cooled down the first dorsal interosseous muscle of human subjects down to 10°C. Subjects were asked to perform isometric constant-force abduction contractions of the index finger at 20% and 80% MVC. The relationship of the initial median frequency percentage, %*Iv*_*med*_, versus intramuscular temperature was found to be linear with sensitivities (slopes) of 3.03%/°C and 3.48%/°C for 20% and 80% MVC, respectively [11]. Since %*Iv*_*med*_ is given by 100*dT*/(*T*_*MI*_−*T*_M0_). Therefore, using the reference temperature value of 33°C as in [11] and absolute initial temperature of 0°C, we obtain a sensitivity or slope of 3.03%/°C, consistent with the experimental results [20].

**Table 5:** Person correlation and beta values for predicting the initial percentage median frequency %*Iv*_*med*_ from parameters *S*,*h*,*a*, and *dT*.

Person correlation coefficients						Linear model
	Fit parameters			Fit parameters	%*Iv*_med_	*β* values
Fit parameters	*dT*	*a*	*S*			
*S*				*S*	0.517	-0.021
*a*			0.001	*a*	0.206	-0.157
*dT*		0.051	0.552	*dT*	0.969*	0.973**

Shows Person correlation and beta values obtained from multiple linear regression analysis. Only *dT* had a significant (*p < .01) Person correlation with %*Iv*_*med*_ and only *dT* predictor had significant (**p <0.01) effect in the full model. The predictor model was able to account for 96.5% of the variance in %*Iv*_*med*_ *F(3*,*8)* = 45.63, *p*<0.001, *R* ^*2*^ = 0.965.

#### *v*_*med*_ vs. *c*_*m*_

**Fig 5:**
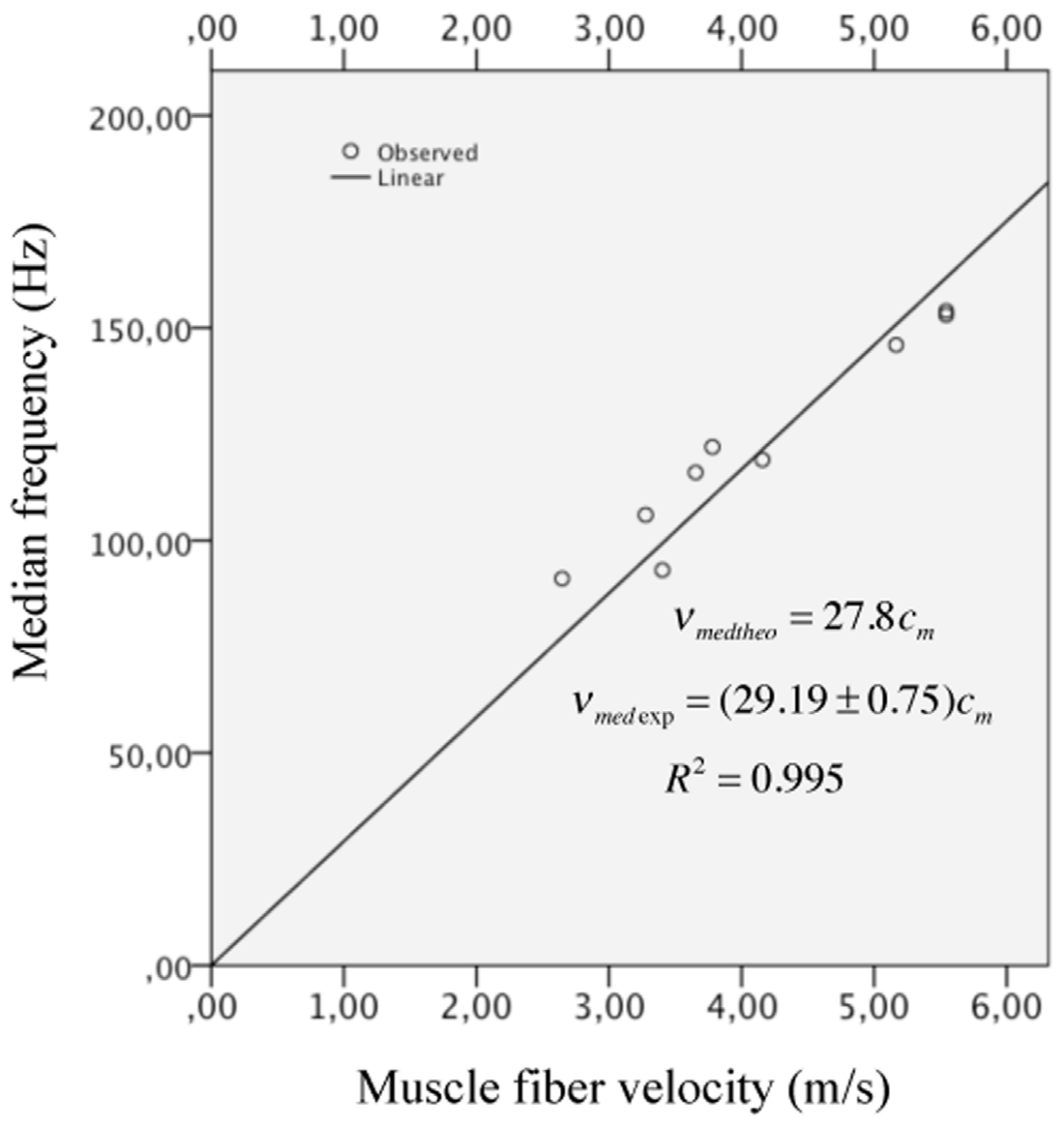
Relationship between medium frequency *v*_*med*_ and parameter muscle fiber velocity *c*_*m*_. The slope value of 29.19±0.75 *Hz*/*m*/*s* represents a difference of less than 5% with respect to the theoretical value of 27.80 *Hz*/*m*/*s*.

Multiple linear regression analysis was used to develop a model for predicting *v*_*med*_from parameters *S*, *h*, *a*, and *c*_*m*_. Table 6 shows the results of these analyses. Only *c*_*m*_ had a significant (*p* < .01) Pearson correlation with *v*_*med*_ and only *c*_*m*_ predictor had a significant effect (*p* <0.01) in the full model. The predictor model was able to account for 96.5% of the variance in *v*_*med*_, *F* (3,8) = 45.63, *p*<0.001, *R* ^*2*^ = 0.965. As a consequence of this result, we performed a linear fit between *v*_*med*_ and *c*_*m*_ only. We found a slope value of 29.19±0.75 *Hz*/*m*/*s*. This result supports the theoretical equation *v*_*med*_ = 3.503*c*_*m*_/2*l*. If we use in *v*_*med*_ = 3.503*c*_*m*_/2*l* the average value for the gastrocnemius medial fiber length of 6.3±1.2 cm as reported in [22], we obtain a slope value of 27.80 *Hz*/*m*/*s*, which is of the same order of magnitude as 29.19 *Hz*/*m*/*s*. The experimental data and the linear fit are shown in Fig 5. Now, by introducing the experimental fiber length value of 6.8 cm for the Tibialis anterior muscle fiber length [26] in the expression *v*_*med*_ = 3.503*c*_*m*_/2*l* we obtained *v*_*med*_ = (25.8*Hz*/*m*/*s*)*c*_*m*_. In reference [9] the experimental relationship found is *v*_*med*_ = (23.1*Hz*/*m*/*s*)*c*_*m*_ for the same muscle, which is of the same order of what we found theoretically.

**Table 6:** Person correlation and beta values for predicting the median frequency *v*_*med*_ from parameters *S*,*h*,*a*, and *c*_*m*_.

Person correlation coefficients						Linear model
	Fit parameters			Fit parameters	*v*_med_	*β* values
Fit parameters	*c*_*m*_	*a*	*S*			
*S*				*S*	0.517	-0.02
*a*			0.001	*S*	0.206	-0.151
*c*_*m*_		0.057	0.552	*dT*	0.970*	0.973**

Shows Person correlation and beta values obtained from multiple linear regression analysis. Only *c*_*m*_ had a significant (*p < .01) Pearson correlation with *v*_*med*_ and only *c*_*m*_ predictor had significant (**p <0.01) effect in the full model. The predictor model was able to account for 96.5% of the variance in *v*_*med*_ *F* (3,8) = 45.63, *p*<0.001, *R* ^*2*^ = 0.965.

#### *I* vs. *dT*

**Fig 6:**
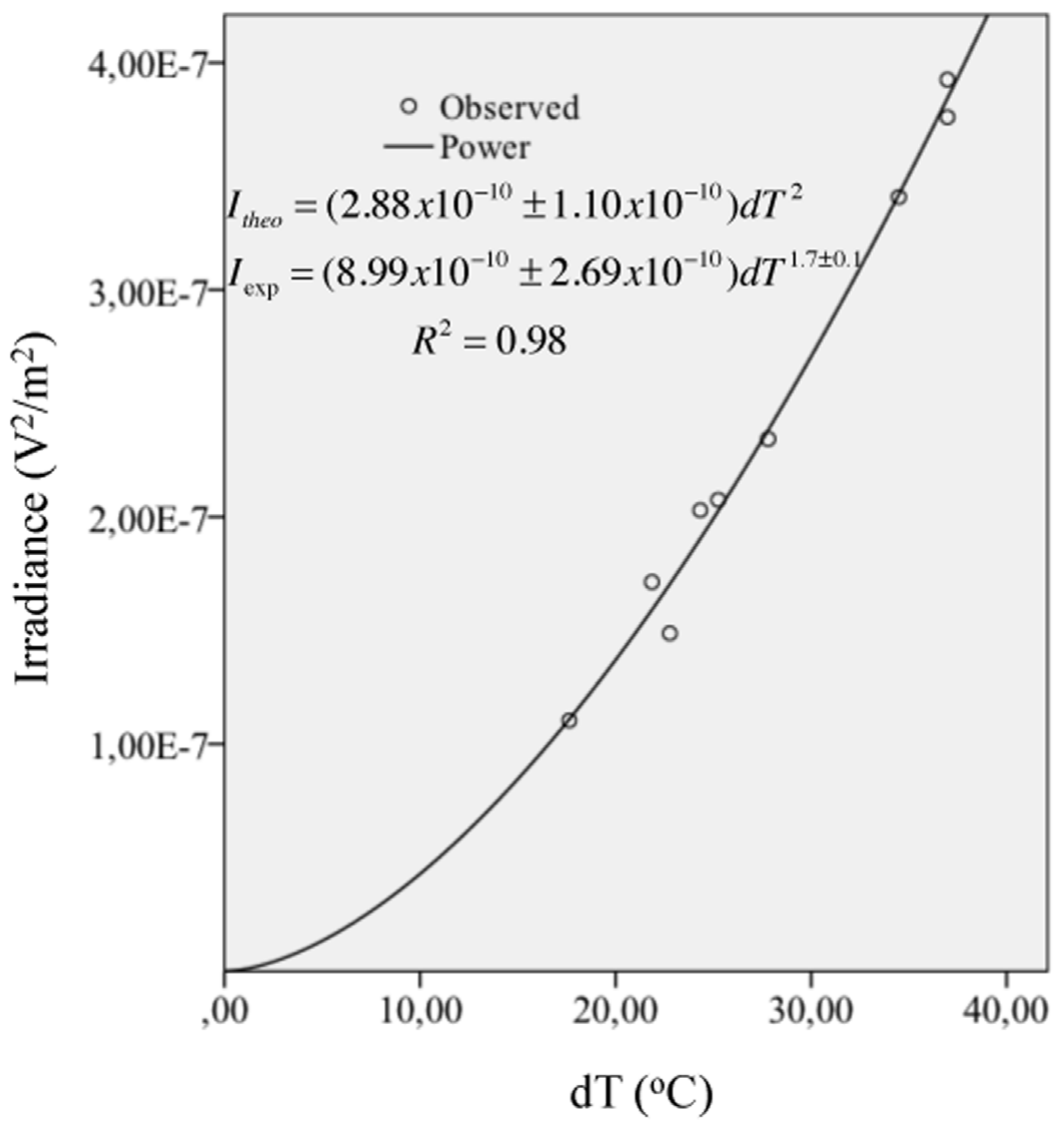
Experimental irradiance result. The exponent value is 1.7±0.1, which has a difference of 15% with respect of the theoretical value of 2.

Multiple linear regression analysis was used to develop a model for predicting the logarithm of irradiance from the logarithm of parameters *S*, *h*, *a*, and *dT*, Table 7 shows the results of these analyses. Only *dT* had a significant (*p* < .01) Person correlation with irradiance and only the *dT* and *a* predictors had significant (*p* < .05) partial effects in the full model. The predictor model was able to account for 99.5% of the variance in irradiance, *F(3*,*8)* = 364.66, *p* <0.001, *R* ^*2*^ = 0.995. This result supports Eq 2. Based on these results and Eq 2 we performed a power fit with the product *adT* as independent variable and obtained the relationship *I* = (8.99×10^−10^±2.69×10^−10^)*dT* ^1.7±0.1^, *F(3*,*8)* = 372.58, *p* < 0.001, *R* ^*2*^ = 0.982. From Eq 2 we can see that the fit exponent is close to the theoretical value of 2 and the constant value can be obtained from *hπ* ^5^ *a* ^2^/60*l* ^2^ (see Eq 2). Substituting the fitted parameters *h*, *a* and the average value for the gastrocnemius medial fiber length of 6.3±1.2 cm as reported in [22] we obtained a value of 2.88×10^−10^±1.10×10^−10^, which is of the same order of magnitude as 8.99×10^−10^. Fig 6 shows the experimental data and the power fit.

**Table 7:** Person correlation and beta values for predicting the logarithm of irradiance *Log*(*I*)from the logarithm of parameters*S*,*h*,*a*, and *dT*.

Person correlation coefficients						Linear model
	Fit parameters			Fit parameters	*Log*(*I*)	*β* values
Fit parameters	*Log*(*dT*)	*Log*(*a*)	*Log*(*S*)			
*Log*(*S*)				*Log*(*S*)	0.565	0.29
*Log*(*a*)			0.001	*Log*(*a*)	0.149	0.10**
*Log*(*dT*)		0.051	0.552	*Log*(*dT*)	0.993*	0.972**

Shows Person correlation and beta values obtained from multiple linear regression analysis. Only Log()*dT* had a significant (*p < .01) Person correlation with Log(irradiance) and only the Log(*dT*) and Log(*a*) predictors had significant (**p < .05) partial effects in the full model. The predictor model was able to account for 99.5% of the variance in irradiance, *F(3*,*8)* = 364.66, *p* <0.001, *R* ^*2*^ = 0.995

### Comparison between experimental studies on muscle PH, aging and fatigue vs. theoretical relationships inferred from Planck’s distribution (VI)

#### *Iv*_*med*_ and *Ic*_*m*_ vs. muscle PH

**Fig 7:**
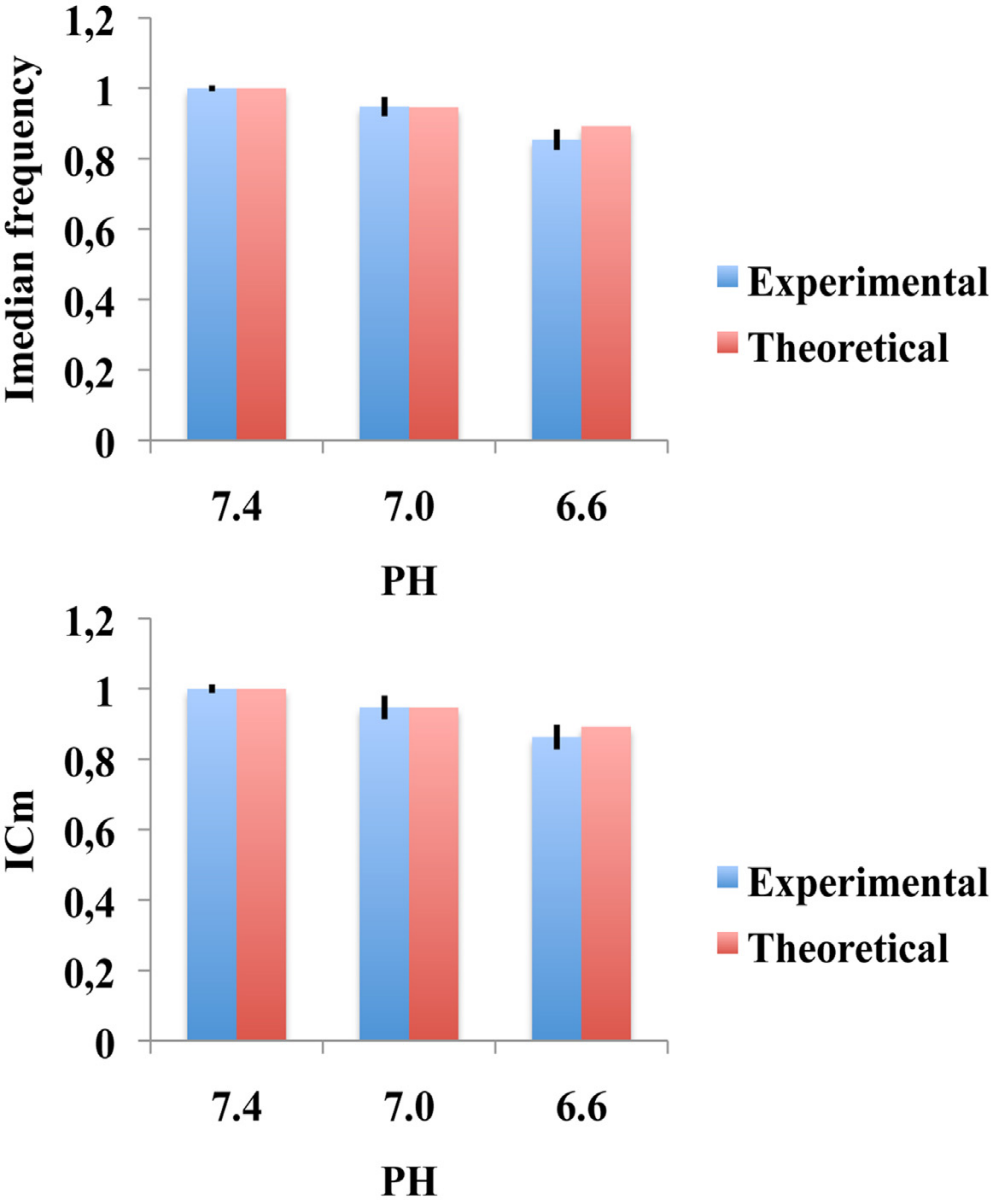
Theoretical and experimental comparison between initial median frequency values and initial muscle fiber velocity values when the muscle PH changes. The theoretical values are similar to the experimental values found in [14].

Experimentally the initial median frequency *Iv*_*med*_ decreased from 1.000±0.008 (PH 7.4) to 0.948±0.027 (PH 7.0) to 0.854±0.029 (PH 6.6) [14]. The initial muscle fiber velocity *Ic*_*m*_, calculated similarly to *Iv*_*med*_, decreased from 1.000±0.012 (PH 7.4) to 0.947±0.033 to 0.863±0.035 (PH 6.6) [14]. The plot of the experimental values *Iv*_*med*_ vs. *Ic*_*m*_ gave a straight line with slope of 1.07 (r>0.99) [14]. Now, in methods we showed that.*Iv*_*med*_ = *Ic*_*m*_ = *PH*/*PH*_*I*_. This last expression tells us that we should expect a linear relationship between *Iv*_*med*_and *Ic*_*m*_with slope value of 1. A value of 1.07 was found in reference [14]. If we take *PH*_*I*_ = 7.4 (as in reference [14]), we get *Iv*_*med*_ = *Ic*_*m*_ = 1 (7.4/7.4), *Iv*_*med*_ = *Ic*_*m*_0.946 (7.0/7.4), *Iv*_*med*_ = *Ic*_*m*_ = 0.892 (6.6/7.4). These theoretical values are similar to the experimental values found in [14] as shown in Fig 7.

#### *v*_*med*_ and *c*_*m*_ vs. muscle aging

We have shown in methods that the relative change for the *BMR* is given by the addition of the *BMR* per surface area (*BMRS*) relative change plus the body surface (*BSA*) relative change, or *δBMR*/*BMR* = *δBMRS*/*BMRS*+*δBSA*/*BSA*. Firstly, the relative change of *BSA* due to aging can be estimated as follows: The body surface *BSA* formula is given by [17] *BSA*(*cm* ^2^) = *WEIGHT* ^0.425^(*Kg*)×*HEIGHT* ^0.725^(*cm*)×71.84. We can estimate the value of *δBSA*/*BSA* by using anthropometry data from [22], where fifteen healthy young men [(age: 25.3 ± 4.5 years (range 19–35 years), height: 176.4 ± 7.7 cm, mass: 79.1 ±11.9 kg, mean ±SD] and 12 healthy elderly men [age: 73.8 ±4.4 years (range 70–82 years), height: 174.1 ±4.4 cm, mass: 76.8 ± 7.6 kg] participated in a sarcopenia and aging study. From these data we obtained that *δBSA*/*BSA* = 0.02 (see S1 File). Secondly, in a recent study [18], the neuromuscular adaptation in young and aged subjects was evaluated by following the changes of *v*_*med*_ and *c*_*m*_ of the EMGA PSD while the subjects performed maximal voluntary contractions of the Tibialis anterior muscle. The subjects were 13 healthy older females (70.8 ± 3.1 years old) and 12 healthy young females (21.4 ±1.7 years old). The *BMR* per surface area value is 41,1W/m^2^ for 21 year-old females and 35,9 W/m^2^ for 71 year-old females [17]. Assuming that approximately 20% of these BMRS corresponds to the muscles, that gives 8.22 W/m^2^ and 7.18 W/m^2^ respectively. From these data, we obtain *δBMRS*/*BMRS* = (8.22−7.18)/8.22 = 0.13. Finally by adding both relative changes, we get *δBMR*/*BMR* = 0.15. From methods section we know that *δBMR*/*BMR* = *δE*_*TH*_/*E*_*TH*_, where *E*_*TH*_ is the muscle thermal energy and from there we found that *δE*_*TH*_/*E*_*TH*_ = *δdT*/*dT* = *δv*_*med*_/*v*_*med*_ = *δc*_*m*_/*c*_*m*_. Therefore we should expect a decrease of the order of 15% on the median frequency and the muscle fiber velocity for the older subjects. The reported averaged median frequency peak, for young subjects, was 63.68 ± 7.15 Hz and the muscle fiber velocity was 4.73 ± 0.99 m/s. A decrease of 15% gives the value of 54.13± 6.08 Hz for the median frequency and 4.02 ± 0.84 m/s for the muscle fiber velocity, both results are within the experimental interval values of 52.29 ± 7.52 Hz and 3.85 ± 0.56 m/s reported in [18].

#### *v*_*med*_, *c*_*m*_ and the PSD amplitude evaluated at *v*_*med*_ vs. muscle fatigue

**Fig 8:**
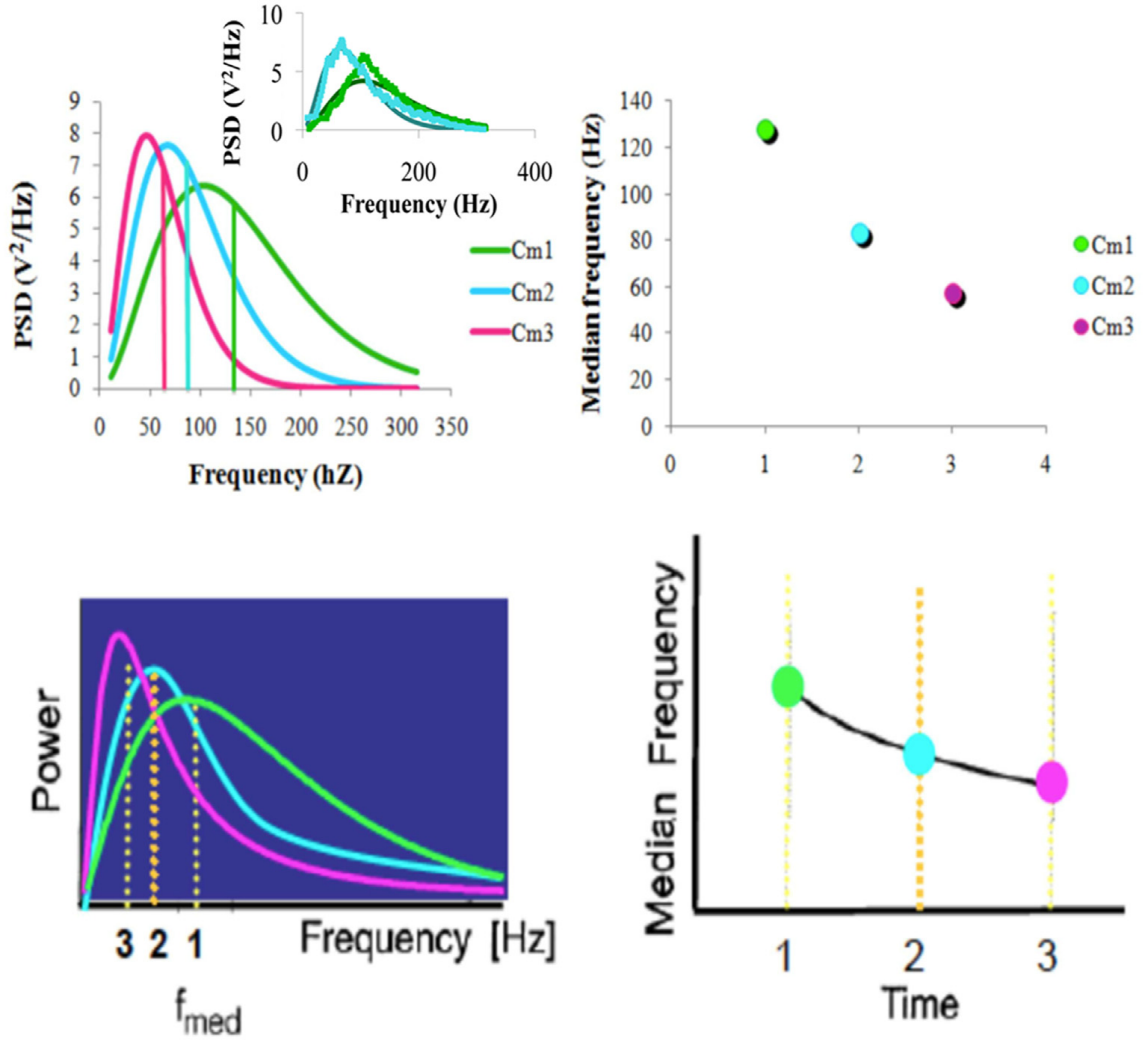
Fatigue effect results compared with the model present here (top row) and a scheme extracted from references [1, 13] (low row). The model curves were fitted with parameters *c*_m1_ = 4.539 m/s, *c*_m2_ = 2.179 m/s, *c*_m3_ = 1.226 m/s and *v*_01_ = 36.6 Hz, *v*_02_ = 23.87 Hz, and *v*_03_ = 16.47Hz. The insert shows experimental results and their fit at the beginning and at the end of a constant-force muscle contraction.

As it was discussed in the methods section, fatigue effects can be described by the Planckian distribution when *δv*_*med*_≤0, *δc*_*m*_≤0 and 3|*δv*_*med*_/*v*_*med*_|≤−2*δc*_*m*_/*c*_*m*_, where *δv*_*med*_, and *δc*_*m*_ represent the physiological changes on the median frequency and velocity due to fatigue. With these conditions, *v*_*med*_ and *c*_*m*_ decreases, the EMGA PSD evaluated at *v*_*med*_ should increase and the PSD should shift towards low frequencies as well due to a sustained contraction of the muscle. Fig 8 shows the comparison between experimental data extracted from [1, 13] and Eq 1. As can be seen in the Fig 8, the Planckian distribution provides a very good fit to the experimental results.

## Discussion and Conclusions

From all the above results we can conclude that the power spectral density of isometric contractions in muscle fibers can be described by a Planckian distribution. As we mention earlier in the introduction, Planck’s distribution describes the electromagnetic radiation emitted by a black body in thermal equilibrium at a definite temperature and the radiated energy can be considered to be the product of standing waves or resonant modes of the radiating cavity.

The standing waves in muscle fibers can be related to resonant modes in a radiative cavity through the phenomenon known as the size principle in muscles [27–29]. The size principle describes the observation that, as more force is demanded, MUs are recruited in a specific order according to the amplitude of their force output. Henneman's group [27–29] first showed that the size of a MU is proportional to the amount of muscle fibers it innervates. They also found that larger impulses represented the firing of larger MUs, and that the smallest MUs represented by the smallest impulse amplitude fired first and had lower thresholds for stretch, while larger MUs fired last and had higher thresholds [30]. Another demonstration of this principle was shown in a study of 78 subjects asked to perform isometric maximal voluntary contractions of the Quadriceps femoris muscle [31]. Results showed that as the force generated by the muscle increased, the MUAP's amplitude and firing frequency increased, while the number of recruited MUs decreased. This indicates that slower, low force MUs were being recruited first, while fast, high force MUs were being recruited later.

**Fig 9:**
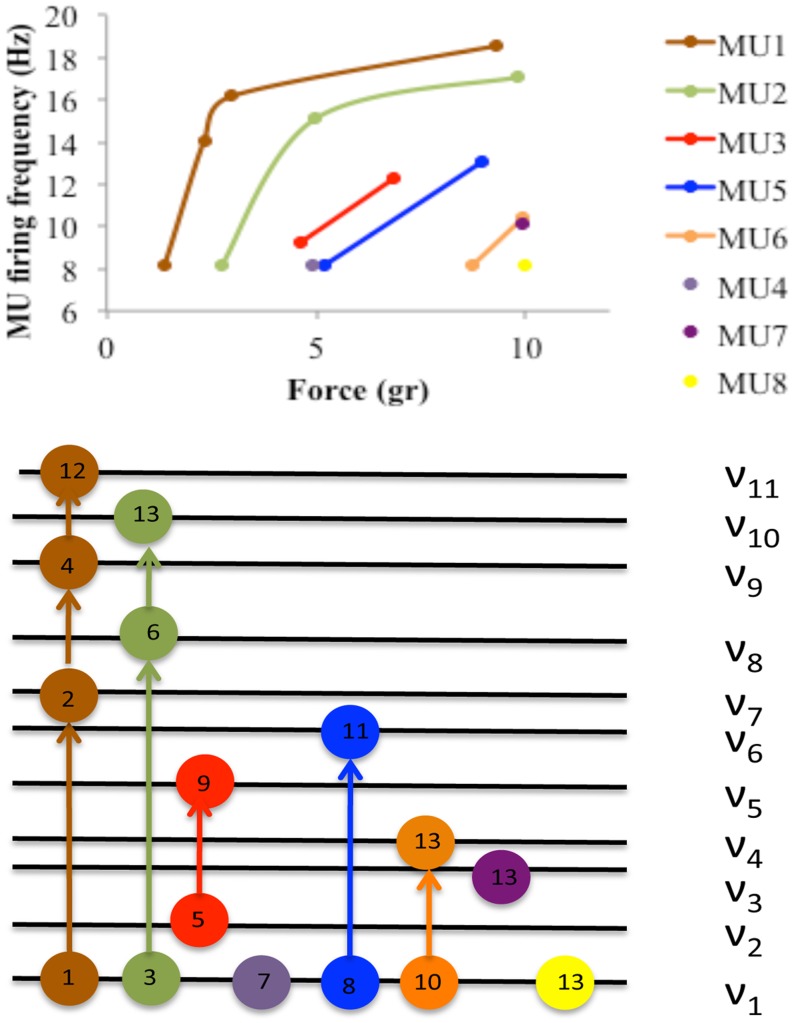
(top). MU firing frequencies versus the force measured with a strain gauge assembly placed perpendicular to the dorsal surface of the middle finger on the distal end of the first phalanx. As more force is exerted a particular MU can increase its firing rate from a low firing rate or more MUs are solicited at low firing rate. Only 8 MUs of 60 are shown (adapted from [32]). (Bottom) Frequency diagram for the 8 MUs shown at the top. In this diagram every MU can be seen as an oscillator with different frequency levels *v*_*i*_. Here we can see all the transitions involved up to a force of 10 gram-force. The numbers on the circles indicate the temporal sequence for the appearance of each transition. The frequency *v*_*i*_ ranges from 8 Hz up to 19 Hz.

We propose that the firing frequency of any given MU can be thought of as an electromagnetic resonant mode, whose energy is proportional to its firing frequency. A muscle can be likened to a radiating cavity, where its set of frequency transitions is defined by the resonant modes of its MUs. Just as in the radiating body case, where the probability of exciting the upper modes is less likely, it is less probable to excite MU with high frequencies. Fig 9 demonstrates this relationship. Fig 9 (top) shows the firing-rate behavior of 8 motor units as a function of the size of an isometric voluntary contraction from an experiment conducted with 60 motor units by Monster and Chan [32]. The isometric voluntary contraction level is represented as force measured with a strain gauge assembly placed perpendicularly to the dorsal surface of the middle finger on the distal end of the first phalanx. The activation of 8 motor units shown in Fig 9 (top) represent a force of 10 gram-force. As more force is exerted, either a single MU increases its firing rate to higher levels or, alternatively, additional slow-firing MUs are solicited. The diagram in Fig 9 (bottom) represents the data from Fig 9 (top) in terms of resonant modes. The force of 10 gram-force can be described as having 11 frequency levels *v*_*i*_ and every MU can be seen as an oscillator that reaches different frequency levels, where each frequency level represents a resonant mode. Thus, the first MU (brown) makes 4 transitions from level *v*_1_ to *v*_11_, a second MU (green) makes 3 transitions, the third MU (red) only makes two transitions, etc. The numbers on the circles indicate the temporal sequence for the appearance of each transition. Following the blackbody analogy, we can hypothesize that the energy should be proportional to the respective characteristic oscillation frequency *v* of the oscillator.

In summary, the fact that a Planckian distribution successfully describes many physiological relationships that involve physiological variables and the fitting parameter *dT* means that the MUAPs may be seen as electromagnetic resonant modes confined at thermal equilibrium inside the muscle, where they form a system of standing waves and the energy of each mode is proportional to its frequency. The temperature range where we expect the Planckian distributions can be used is from 10 up to 37°C. The size principle establishes that as the force generated in a muscle increases, the MUAP's amplitude and its firing frequency increase, meaning that MUs are being recruited from slow, low force to fast, high force with the number of MUs decreasing as the frequency increases. Moreover, the individual MU frequency response is not continuous but is rather a series of discrete values. Every discrete value should represent a mode with energy proportional to *hv*. The current results show that it is less probable to excite upper MU frequencies or modes because the probability of occupying these modes is not the same and requires an extra energy proportional to *hv*, making the process for exciting upper modes less probable, just like the Planckian distribution predicts. It is interesting to note that from thermodynamical arguments, Eq 1 represents a body that radiates the largest quantity of energy per frequency [33].

Finally the Planckian distribution needs five parameters named *S*, *h*, *a*, *l* and *dT*. Our fitting and multiple regression analyses revealed that *h* and *a* can be considered as constants, because of the level of variance they presented compared with the rest of parameters. The constant *h* value we obtained here is 1.59×10^−13^ V^2^/Hz^2^ but it can be estimated from references [31] *h* = 2.759×10^−13^±0.347×10^−13^ and [32] *h* = 10.48×10^−13^±1.92×10^−13^ (see S1 File and S1 Fig). Both values are of similar order of magnitude as the value we estimated with our experimental data. The parameter *l* value can be determined either by the fitting process or by using its averaged value, given that it represents the fiber length. Here we have followed the latter approach because averaged fiber lengths are well known for all human muscles. The parameter *dT* can be measured by using a thermocouple needle inside the muscle or can also be obtained from the fitting process. Only the parameter *S* cannot be measured directly and needs to be inferred from the fitting process.

In conclusion, the current study uncovers the physical fundamentals of isometric muscle contractions. This is important to the scientific community because considering MUAPs as electromagnetic resonant modes confined at thermal equilibrium inside the muscle not only accounts for previously known theoretical relationships, but also provides explanations to previously observed phenomena and makes novel predictions. However, more elaborate theoretical models would be needed to explain more complex movements such as dynamic contractions and gait.

## Supporting Information

S1 FigThe relationship of ${\langle A\rangle }_{Tj}^{2}$ versus $N_{Tj}{\langle \nu \rangle }_{Tj}^{2}$ values.The slope value is *h* = 2.759×10^−13^±0.347×10^−13^ V^2^/Hz^2^. The error bars represent the propagation of uncertainty via algebraic manipulations of the individual standard errors in the firing frequency and amplitude for each force level from data presented in [32].(TIF)

S1 File This file contains the whole mathematical details that were used to develop all the physical relationships shown in here. The file is organized in sections similar to the main manuscript to help readers finding better the information.
(DOCX)
