# Supplementary figures and images for: Planckian Power Spectral Densities from Human Calves during Posture Maintenance and Controlled Isometric Contractions
Source: PLoS One. 2015 Jul 27;10(7):e0131798. doi: 10.1371/journal.pone.0131798 (PMC4516241; doi:10.1371/journal.pone.0131798)

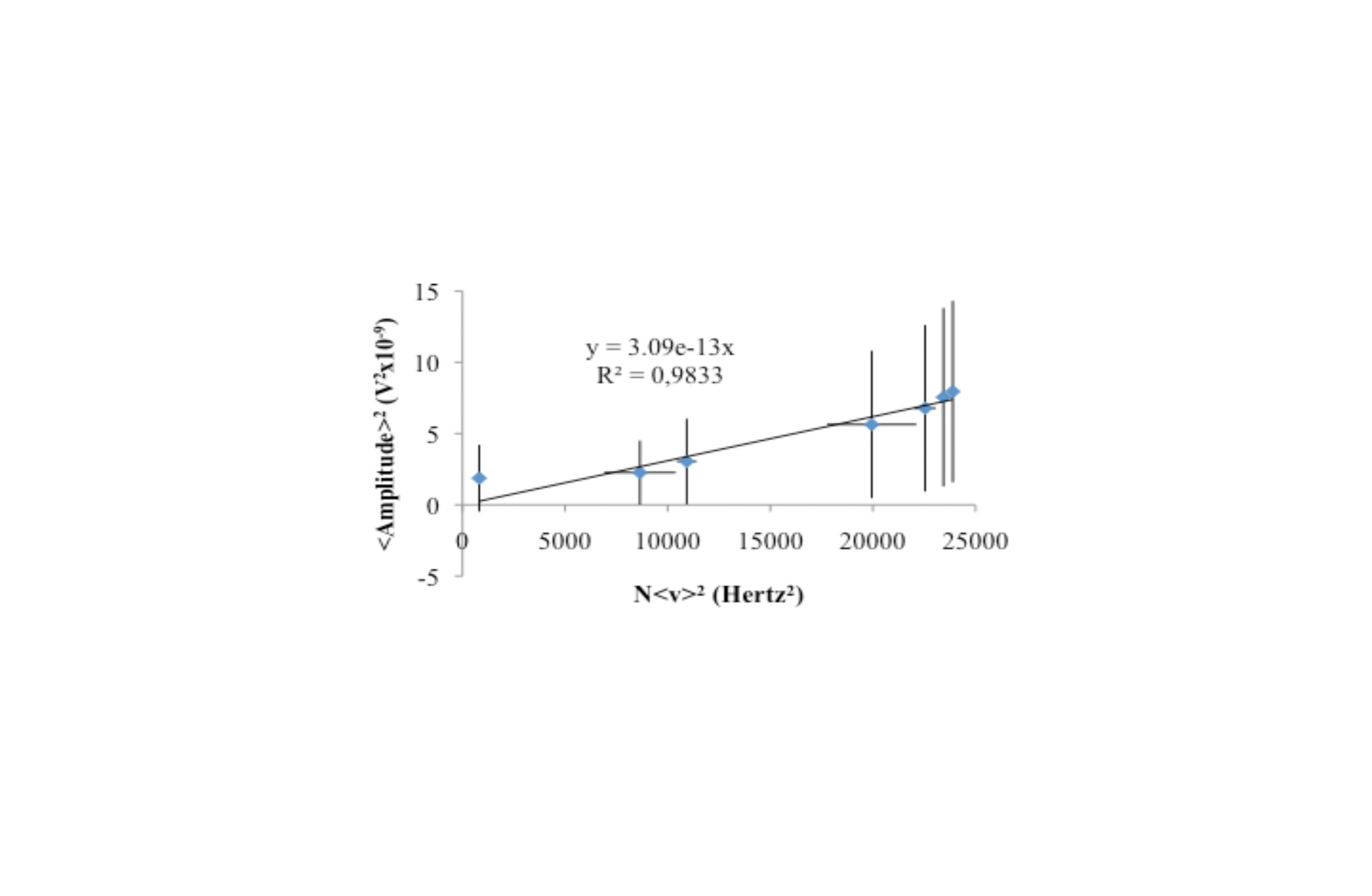

Supplement: S1 Fig — The slope value is h = 2.759×10^−13^±0.347×10^−13^ V2/Hz2. The error bars represent the propagation of uncertainty via algebraic manipulations of the individual standard errors in the firing frequency and amplitude for each force level from data presented in [32]. (TIF) [file pone.0131798.s001.tif]
