# Supplementary material for: Planckian Power Spectral Densities from Human Calves during Posture Maintenance and Controlled Isometric Contractions
Source: PLoS One. 2015 Jul 27;10(7):e0131798. doi: 10.1371/journal.pone.0131798 (PMC4516241; doi:10.1371/journal.pone.0131798)
Supplement: S1 File — The file is organized in sections similar to the main manuscript to help readers finding better the information. (DOCX) [file pone.0131798.s002.docx]

**Supplementary information**

J.E Lugo^1*^, R. Doti^1¶^, and J. Faubert^1¶^

^1^Visual psychophysics and perception laboratory, School of Optometry, Université de Montréal, Montréal, Quebéc, Canada

* Corresponding author

E-mail: [eduardo.lugo@gmail.com](mailto:eduardo.lugo@gmail.com)

^¶^,These authors contributed equally to this work.

**General**

All the averaged power spectral densities were fitted by means of equations of the form:

$P(\nu,\nu_{0})=\frac{Sh\pi}{c_{m}^{2}}\frac{\nu^{3}}{e^{\frac{\nu}{\nu_{0}}}-1}=\frac{Sh\pi}{(2l\nu_{0})^{2}}\frac{\nu^{3}}{e^{\frac{\nu}{\nu_{0}}}-1}$, (1)

where $\nu_{0}=adT$ $h$, $S$, $l$ are fitting parameters and $\nu$ is the linear frequency.

**Maximum frequency**

Now, we can calculate the frequency that maximizes equation (1) by taking its derivative and equating it to zero. The final equation is:

$\frac{\nu_{\max}}{\nu_{0}}=3\frac{e^{\frac{\nu_{\max}}{\nu_{0}}}-1}{e^{\frac{\nu_{\max}}{\nu_{0}}}}$, (2)

Equation (2) can be rewritten as:

$\frac{\nu_{\max}}{\nu_{0}}=C$, (3)

where $C$ is a constant that is found by solving numerically equation (2). Its value is 2.821.

**Median frequency**

To compute the median frequency we need to calculate the total EMGA power for a given $\nu_{0}$ over a finite range of frequencies, say from $\nu_{1}$ to $\nu_{2}$, it is necessary to compute the integral:

$P(\nu_{1}-\nu_{2},\nu_{0})=\int_{\nu_{1}}^{\nu_{2}} P\left(\nu,\nu_{0}\right)d\nu=\int_{\nu_{1}}^{\infty} P\left(\nu,\nu_{0}\right)d\nu-\int_{\nu_{2}}^{\infty} P\left(\nu,\nu_{0}\right)d\nu$. (4)

So, we need to solve the following integral

$P(\nu-\infty,\nu_{0})=\int_{\nu}^{\infty} P\left(\nu,\nu_{0}\right)d\nu=\int_{\nu}^{\infty} \frac{A_{p}\nu^{3}}{e^{\frac{\nu}{\nu_{0}}}-1}d\nu$. (5)

Multiplying both numerator and denominator of the right-hand side of equation (5) by $e^{-\frac{\nu}{\nu_{0}}}$ gives

$P(\nu-\infty,\nu_{0})=\int_{\nu}^{\infty} \frac{A_{p}\nu^{3}e^{-\frac{\nu}{\nu_{0}}}}{1-e^{-\frac{\nu}{\nu_{0}}}}d\nu$. (6)

We know that

$\frac{1}{1-r}=\sum_{n=0}^{\infty} r^{n}\begin{matrix} \end{matrix}for\begin{matrix} \end{matrix}r^{2}<1$, (7)

because $\nu$ and $\nu_{0}$ are always greater than zero then $r=e^{-\frac{\nu}{\nu_{0}}}<1$. Equation (6) then can be written as:

$P(\nu-\infty,\nu_{0})=A_{p}\int_{\nu}^{\infty} \nu^{3}\left(\sum_{n=1}^{\infty} e^{-\frac{n\nu}{\nu_{0}}}\right)d\nu$. (8)

As compared with series $e^{-n}$, the series under the integral is uniformly and absolutely convergent; therefore, the integration can be performed term by term, and equation (8) becomes

$P(\nu-\infty,\nu_{0})=A_{p}\sum_{n=1}^{\infty} \int_{\nu}^{\infty} \nu^{3}e^{-\frac{n\nu}{\nu_{0}}}d\nu$. (9)

Integrating by parts the integral inside the summation, in equation (9) we obtain:

$P(\nu-\infty,\nu_{0})=A_{p}\sum_{n=1}^{\infty} \frac{\nu_{0}^{4}e^{-\frac{n\nu}{\nu_{0}}}}{n^{4}}\left[\left(\frac{n\nu}{\nu_{0}}\right)^{3}+3\left(\frac{n\nu}{\nu_{0}}\right)^{2}+6\left(\frac{n\nu}{\nu_{0}}\right)+6\right]$. (10)

Now, we can set up $\nu=0$ in equation (10) to obtain the total power over all frequencies

$P(0-\infty,\nu_{0})=6A_{p}\nu_{0}^{4}\sum_{n=1}^{\infty} \frac{1}{n^{4}}=6A_{p}\nu_{0}^{4}\frac{\pi^{4}}{90}=\frac{A_{p}\pi^{4}\nu_{0}^{4}}{15}$. (11)

If we divide equation (11) by the EMGA emission surface then we obtain the EMGA irradiance as:

$I(0-\infty,\nu_{0})=\frac{A_{p}\pi^{4}\nu_{0}^{4}}{15A}$, (12)

which is independent of the emission surface.

Dividing (10) by (11) gives the normalized total intensity that ranges from 0 to 1

$P\left(\frac{\nu}{\nu_{0}}\right)_{Norm}=\frac{15}{\pi^{4}}\sum_{n=1}^{\infty} \frac{e^{-\frac{n\nu}{\nu_{0}}}}{n^{4}}\left[\left(\frac{n\nu}{\nu_{0}}\right)^{3}+3\left(\frac{n\nu}{\nu_{0}}\right)^{2}+6\left(\frac{n\nu}{\nu_{0}}\right)+6\right]$. (13)

The median frequency is defined as the EMGA frequency where the intensity is half of the total power. In other words, it is the frequency where equation (13) equals 0.5, then the median frequency can be found by solving numerically the following equation:

$\frac{\pi^{4}}{30}=\sum_{n=1}^{\infty} \frac{e^{-\frac{n\nu_{med}}{\nu_{0}}}}{n^{4}}\left[\left(\frac{n\nu_{med}}{\nu_{0}}\right)^{3}+3\left(\frac{n\nu_{med}}{\nu_{0}}\right)^{2}+6\left(\frac{n\nu_{med}}{\nu_{0}}\right)+6\right]$, (14)

We can approximate the infinite summation for a finite one by assuming we want a specific accuracy on the result, for instance if we only use two terms from the infinite summation we obtain the equation

$\begin{matrix} \frac{\pi^{4}}{30}=\frac{e^{-\frac{\nu_{med}}{\nu_{0}}}}{1^{4}}\left[\left(\frac{\nu_{med}}{\nu_{0}}\right)^{3}+3\left(\frac{\nu_{med}}{\nu_{0}}\right)^{2}+6\left(\frac{\nu_{med}}{\nu_{0}}\right)+6\right]+ \\ \frac{e^{-\frac{2\nu_{med}}{\nu_{0}}}}{2^{4}}\left[8\left(\frac{\nu_{med}}{\nu_{0}}\right)^{3}+12\left(\frac{\nu_{med}}{\nu_{0}}\right)^{2}+12\left(\frac{\nu_{med}}{\nu_{0}}\right)+6\right]+E\left(\frac{3\nu_{med}}{\nu_{0}}\right) \end{matrix}$, (15)

where the function E represents the error. Solving (15) numerically gives an approximate value of

$\frac{\nu_{med}}{\nu_{0}}=3.503$. (16)

**Mean frequency**

The mean frequency can be calculated as follows:

$\nu_{mean}=\frac{\int_{0}^{\infty} \nu P\left(\nu,\nu_{0}\right)d\nu}{\int_{0}^{\infty} P\left(\nu,\nu_{0}\right)d\nu}=\frac{\int_{0}^{\infty} \frac{A_{p}\nu^{4}}{e^{\frac{\nu}{\nu_{0}}}-1}d\nu}{P(\nu_{0})_{0-\infty}}$, (17)

with

$\int_{0}^{\infty} \frac{A_{p}\nu^{4}}{e^{\frac{\nu}{\nu_{0}}}-1}d\nu=A_{p}24Z\left(5\right)\nu_{0}^{5}$, (18)

where $Z(n)$ is the Riemann function. By using equation (11) and (18) then equation (17) transforms:

$\nu_{mean}=\frac{24Z\left(5\right)}{\frac{\pi^{4}}{15}}\nu_{0}$, (19)

Since $Z(5)=1.037$ then

$\nu_{mean}=3.832\nu_{0}$, (20)

**Bandwidth**

The bandwidth can be calculated by normalizing equation (1) and using $P_{\max}\left(\nu_{\max},\nu_{0}\right)$, we obtain

$\frac{P(\nu,\nu_{0})}{P_{\max}(\nu_{\max},\nu_{0})}=\frac{\frac{A_{p}\nu^{3}}{e^{\frac{\nu}{\nu_{0}}}-1}}{\frac{A_{p}C^{3}\nu_{0}^{3}}{e^{C}-1}}=\left(\frac{\nu}{\nu_{0}}\right)^{3}\frac{e^{C}-1}{e^{\frac{\nu}{\nu_{0}}}-1}\frac{1}{C^{3}}$ , (21)

where we have used equation (3). The two frequencies that delimit the bandwidth are found by solving numerically the equation:

$0.5=\frac{x^{3}}{e^{x}-1}\frac{e^{C}-1}{C^{3}}$ , (22)

where $x=\frac{\nu}{\nu_{0}}$.

From equation (22) we obtain

$\nu_{BW1}=1.16\nu_{0}\begin{matrix} \end{matrix}and\begin{matrix} \end{matrix}\nu_{BW2}=5.41\nu_{0}$. (23)

**Temperature effects on global frequencies**

Now, from equations (3), (16) and (20) we know that in general $\frac{\nu_{j}}{\nu_{0}}=\alpha_{j}$, where $j=\max,\begin{matrix} \end{matrix}\begin{matrix} med,\begin{matrix} \end{matrix} \end{matrix}\begin{matrix} mean. \end{matrix}$ and $\alpha_{j}$ is a constant. Since $\nu_{0}=a(T_{M}-T_{M0})$ then $\nu_{j}=\alpha_{j}a(T_{M}-T_{M0})$ and the sensitivity (slope) is given by the product of constant $a$ and constant $\alpha_{j}$. By taking the $\nu_{jI}$ at temperature $T_{MI}$ as reference, we can calculate the percentage initial maximum, median or mean frequency shift as

$I\nu_{j}\%=100\frac{\nu_{j}}{\nu_{jI}}=100\frac{(T_{M}-T_{M0})}{(T_{MI}-T_{M0})}$ , (24)

and the sensitivity is represented by the slope of equation 24 as follows:

$S_{j}=\frac{100}{(T_{MI}-T_{M0})}.$ (25)

**Intramuscular absolute initial temperature**

The temperature would be the equivalent of the absolute zero lower limit on the thermodynamic temperature scale. However the laws of thermodynamics dictate that absolute zero cannot be reached using only thermodynamic means. Now, the value for can be estimated as follows: Merletti and colleagues [1] cooled down the first dorsal interosseous muscle of human subjects from 37 ^0^C to 10 ^0^C. Subjects were asked to perform isometric constant-force abduction contractions of the index finger at 20% and 80% maximum voluntary contraction (MVC). The initial median frequency of the EMGA-PSD was taken as a reference (measured at 33 ^0^C) and for every degree that the temperature decreased; the measured was normalized by and multiplied by 100 to obtain the initial median frequency percentage, . The relationship of versus intramuscular temperature was a linear relationship (Figure 6 in reference [1]), where the sensitivities (slopes) were 3.03 %/^0^C and 3.48 %/^0^C for 20% and 80% MVC, respectively, and the intercept with the temperature axis was around 0 ^0^C (273.15 *K*). Theoretically, is given by (see above), where is the initial reference temperature and the sensitivity (slope) is then given by . From these results, we can infer that the value for should be close to .That is, as approaches then approaches zero.

**Irradiance vs parameter** $dT$

In order to calculate the irradiance we obtain the total power radiated by a surface of area $S$ from Equation 1 by integrating across the range of possible frequencies i.e.,

, (26)

where , , and is the cutoff frequency with a value of 1 KHz [2,3]. If we assume values for less than 100 Hz, the value of is high of the order of (1000/10) thus Equation 26 can be evaluated to give approximately:

, (27)

and the irradiance is obtained when we divide the total power by the surface of area as

. (28)

**Constant** **value**

Let us go back to Fig. 1 of the manuscript. Given that the constant in Equation 1 has units of or , the product of with the frequency level value has units of . are units of power spectral density, which itself is proportional to energy. Therefore, the energy associated with each frequency level is proportional to the expression . From this hypothesis it is possible to estimate the order of magnitude of as follows: when a subject contracts a muscle to a certain percentage of the maximum voluntary contraction (%MVC) and MUs are activated at frequency level , the total energy associated at this % MVC, should be proportional to . Now, if the subject increases the % MVC and at the end of the extension MUs remain activated, the total energy associated at this new % MVC would be proportional to . If we continue this process up a j% MVC, the total energy associated to j% MVC , would be proportional to or , which can be written as , where the quantity in brackets is the expression for the weighted average firing frequency, with the weights given by the number of MUs activated at the end of each % MVC. Therefore the total energy associated to j% MVC should be proportional to , where is the total number of MUs that were activated from the first % MVC to the j % MVC. Associated with each j % MVC there is also a MUAP amplitude weighted average given by , where represents the MUAP amplitude at the end of the j% MVC. It is also known that for simple waveforms [4], such as the averaged MUAP signal, the average power carried by an acoustic or electromagnetic or by an electrical signal is proportional to the square of the signal's peak amplitude, which in this case equals to . Thus, the average energy transmitted by the signal would be the product of the average power and the interval of time this average power is transmitted. It turns out that this time should be inversely proportional to the firing frequency average . Therefore we can postulate that can also be proportional to the product of and finally we obtain the following relationship:or . Thus, the value of h can be estimated by obtaining the slope of values vs. values.

If we use the size principle arguments discussed in the introduction, we can obtain an estimate of *h* by obtaining the slope of the linear regression analysis on as dependent variable and as the predictor. In reference [5] Conwit and colleagues studied 78 subjects and investigated the relationship of motor unit size, firing rate and force. Subjects were asked to perform isometric MVC of the Quadriceps femoris muscle, from a force level of 5% to 100% MVC (7 levels). The motor population, amplitude and firing frequency for each level of force were measured. This information allowed us to calculate the firing frequency weighted average , the amplitude weighted average and . In S1 Fig. we observe the relationship of versus . The slope value was V^2^/Hz^2^, ,, . The error bars in Figure S1 represent the propagation of uncertainty via algebraic manipulations of the individual standard errors in the firing frequency and amplitude for each force level. Monster and colleagues [6] measured the action potential of 150 units recruited when 4 subjects performed isometric maximal voluntary contractions of the extensor Digitorum communis muscle. The motor population and the amplitude for each level of force (7 levels) were also reported. The only firing frequency information given was for the lowest (8 Hz) and highest (12 Hz) frequency levels. To perform a similar analysis as the one described in S1 Fig. we had to assign the lowest force level to the lowest firing frequency and the highest force level to the highest firing frequency. Intermediate force levels were paired with uniformly distributed firing frequencies values, between 8 and 12 Hz. Again the linear regression analysis for versus had a slope value of V^2^/Hz^2^, ,, . Both values are of similar order of magnitude as the value of V^2^/Hz^2^ estimated with our experimental data (see Table 1 of the manuscript).

**
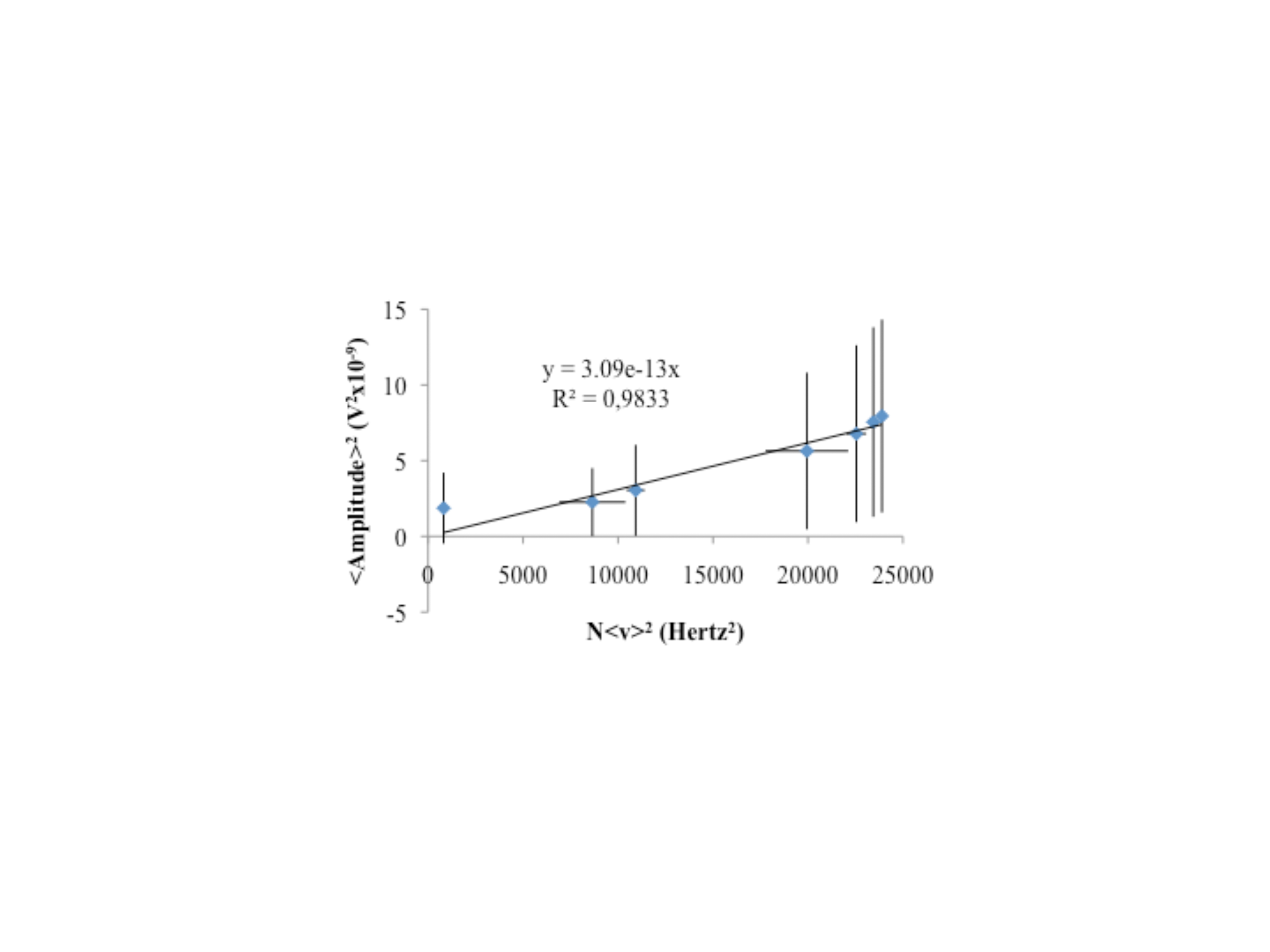
**

**Median frequency $\nu_{med}$ and muscle fiber conduction $c_{m}$ vs muscle PH**

In this case the median frequency can be written as $\nu_{med}=3.503\nu_{0}=3.503a(PH)(T_{M}-T_{M0})$ and the muscle fiber velocity as $c_{m}=2l\nu_{0}=2lc(PH)(T_{M}-T_{M0})$. Where we can notice that parameter $c$ is now a PH function. Now, the initial median frequency and muscle fiber velocity are defined as $I\nu_{med}=\nu_{med}/\nu_{medI}$and $Ic_{m}=c_{m}/c_{mI}$. If the temperature is constant while the PH changes thus $I\nu_{med}=3.503a(PH)(T_{M}-T_{M0})/3.503a(PH_{I})(T_{M}-T_{M0})=a(PH)/a(PH_{I})$ and $Ic_{m}=2la(PH)(T_{M}-T_{M0})/2la(PH_{I})(T_{M}-T_{M0})=a(PH)/a(PH_{I})$. Finally from these equations the result $I\nu_{med}=Ic_{m}=a(PH)/a(PH_{I})$is evident.

**Median frequency , muscle fiber velocity and the PSD amplitude evaluated at the median frequency vs muscle fatigue**

The PSD of the Planckian distribution at the median frequency is

$P_{med}=\frac{A_{p}\nu_{med}^{3}}{e^{\frac{\nu_{med}}{\nu_{0}}}-1}=\frac{\pi Sh\nu_{med}^{3}}{\left[e^{3.503}-1\right]c_{m}^{2}}=\varepsilon\frac{\nu_{med}^{3}}{c_{m}^{2}}$ (29)

where $\varepsilon$ is a constant. Now, during a fatigue task $c_{m}$ changes to $c_{m}'=c_{m}+\delta c_{m}$, $\nu_{med}$ changes to $\nu_{med}^{'}=\nu_{med}+\delta\nu_{med}$ and $P_{med}$ changes to $P_{med}^{'}=P_{med}+\delta P_{med}$ then we can write:

$c_{m}^{'}=c_{m}+\delta c_{m}=c_{m}\left[1+\frac{\delta c_{m}}{c_{m}}\right]$ (30)

and

$\nu_{med}^{'}=\nu_{med}+\delta\nu_{med}=\nu_{med}\left[1+\frac{\delta\nu_{med}}{\nu_{med}}\right]$ (31)

and

$P_{med}^{'}=P_{med}+\delta P_{med}=P_{med}\left[1+\frac{\delta P_{med}}{P_{med}}\right]$ (32)

Now from (29) we have

$\frac{\delta P_{med}}{P_{med}}=\frac{\varepsilon\frac{c_{m}^{2}3\nu_{med}^{2}\delta\nu_{med}-\nu_{med}^{3}2c_{m}\delta c_{m}}{c_{m}^{4}}}{\frac{\varepsilon\nu_{med}^{3}}{c_{m}^{2}}}=\frac{3\delta\nu_{med}}{\nu_{med}}-\frac{2\delta c_{m}}{c_{m}}$ (33)

We can analyze different cases.

i) $\delta\nu_{med}\geq0$ and$\delta c_{m}\geq0$ therefore

$c_{m}^{'}\geq c_{m}$, and $\nu_{med}^{'}\geq\nu_{med}$ This is not known as an experimental result for fatigue effects.

ii) $\delta\nu_{med}\leq0$ and$\delta c_{m}\geq0$ therefore

$c_{m}^{'}\leq c_{m}$, and $\nu_{med}^{'}\geq\nu_{med}$ This is not known as an experimental result for fatigue effects.

iii) $\delta\nu_{med}\geq0$ and $\delta c_{m}\leq0$ therefore

$c_{m}^{'}\geq c_{m}$, and $\nu_{med}^{'}\leq\nu_{med}$ This is not known as an experimental result for fatigue effects either.

iv) $\delta\nu_{med}\leq0$ and $\delta c_{m}\leq0$ therefore

$c_{m}^{'}\leq c_{m}$, and $\nu_{med}^{'}\leq\nu_{med}$ This is known as an experimental result for fatigue effects.

Now we have

$P_{med}^{'}=P_{med}\left[1+\frac{\delta P_{med}}{P_{med}}\right]=P_{med}\left[1+3\frac{\delta\nu_{med}}{\nu_{med}}-2\frac{\delta c_{m}}{c_{m}}\right]$ (34)

Since $3\frac{\delta\nu_{med}}{\nu_{med}}\leq0$u and $-2\frac{\delta c_{m}}{c_{m}}\geq0$ , we have two scenarios

1) $\left|3\frac{\delta\nu_{med}}{\nu_{med}}\right|\geq-2\frac{\delta c_{m}}{c_{m}}$ therefore $P_{med}^{'}\leq P_{med}$ This is not known as an experimental result for fatigue effects either.

2) $\left|3\frac{\delta\nu_{med}}{\nu_{med}}\right|\leq-2\frac{\delta c_{m}}{c_{m}}$ therefore $P_{med}^{'}\geq P_{med}$ This is another experimental result for fatigue effects.

Then fatigue effects due to metabolic changes can be described by the Planckian distribution when $\delta\nu_{med}\leq0$, $\delta c_{m}\leq0$and $\left|3\frac{\delta\nu_{med}}{\nu_{med}}\right|\leq-2\frac{\delta c_{m}}{c_{m}}$.

**Median frequency $\nu_{med}$ and muscle fiber velocity $c_{m}$ vs muscle aging**

We propose that thermal energy$E_{TH}$is proportional to the Basal metabolic rate *BMR*, so any relative change $\delta E_{TH}/E_{TH}$ should be equal to $\delta BMR/BMR$ and because , and therefore . Thus any relative change on the basal metabolic rate should be reflected exactly in the same proportion in and . Since $BMR=(BMRS)BSA$, where $BSA$ is the body surface and $BMR$ is the basal metabolic rate per surface area. Therefore $\delta BMR/BMR=\delta BMRS/BMRS+\delta BSA/BSA$, so the relative change for *BMR* is given by the *BMRS* relative change plus the BSA relative change. The body surface $BSA$ is given by [7]$BSA(cm^{2})=WEIGHT^{0.425}(Kg)xHEIGHT^{0.725}(cm)x71.84$. First, we calculate the BSA for young humans to obtain $BSA_{young}$ then for aged humans to obtain $BSA_{aged}$. The relative change $\delta BSA/BSA$is given by $(BSA_{young}-BSA_{old})/BSA_{young}$. Second, we repeat the same analysis for the $BMRS$ to obtain its relative change given $(BMRS_{young}-BMRS_{old})/BMRS_{young}$.

**Trust Region minimization algorithm**

Let $x\in R_{n}$ be an *n*-dimensional vector of variables, and $\varepsilon(x)=[\varepsilon_{1}(x),...,\varepsilon_{m}(x)]^{T}$ be a *m*-dimensional function of $x$. We are interested in solving the optimization problem

$\min(\sum_{i=1}^{m} \varepsilon^{2}=\left\|\varepsilon(x)\right\|^{2})$ (35)

$$L\leq x\leq U$$

Where, *L* and *U* are lower and upper bounds on the parameter vector *x*. Since the efficient global minimization of Equation (35) for general $\varepsilon(x)$ is an intractable problem, we will have to settle for finding a local minimum. The general strategy when solving non-linear optimization problems is to solve a sequence of approximations to the original problem. At each iteration, the approximation is solved to determine a correction Δ*x* to the vector *x*. For non-linear least squares, an approximation can be constructed by using the linearization $\varepsilon(x+s)\approx\varepsilon(x)+J(x)s$, where the the Jacobian *J*(*x*) of $\varepsilon(x)$ is an *m*×*n* matrix. which leads to the following linear least squares problem:

$$\mathop{Min}\limits_{s} (\left\|J(x)s+\varepsilon(x)\right\|^{2})$$

Unfortunately, naively solving a sequence of these problems and updating $x\to x+s$ leads to an algorithm that may not converge. To get a convergent algorithm, we need to control the size of the step $s$. Depending on how the size of the step $s$ is controlled, non-linear optimization algorithms can be divided into two major categories [8]. The trust region approach approximates the objective function using a model function (often a quadratic) over a subset of the search space known as the trust region. If the model function succeeds in minimizing the true objective function the trust region is expanded; conversely, otherwise it is contracted and the model optimization problem is solved again.

The basic trust region algorithm looks something like this.

1. Given an initial point *x* and a trust region radius $\Delta$.
2. Solve

such that

$$\left\|D(x)s\right\|^{2}\leq\Delta$$

$$L\leq x+s\leq U$$

1. If $\varepsilon(x+s)<\varepsilon(x)$ then $x=x+s$


$$\rho=\frac{\left\|\varepsilon(x+s)\right\|^{2}-\left\|\varepsilon(x)\right\|^{2}}{\left\|J(x)s+\varepsilon(x)\right\|^{2}-\left\|\varepsilon(x)\right\|^{2}}$$

3. if $\rho>0.75$ then $\Delta:=\max\{\Delta,3*\left\|s\right\|\}$
4. else if $\rho<0.25$ then $\Delta:=0.5*\Delta$
5. Go to 2.

Here, $\Delta$ is the trust region radius, *D*(*x*) is some matrix used to define a metric on the domain of $\varepsilon(x)$, $\rho$ is known as the gain ratio. The idea is to increase or decrease the radius of the trust region depending on how well the linearization predicts the behavior of the non-linear objective.

Lower and upper bounds used to fit the Planckian distributions for 9 subjects are presented below :

|  | Lh | Hh | LS | HS | La | Ha | LdT | HdT |
| --- | --- | --- | --- | --- | --- | --- | --- | --- |
| S1 | 1,00E-13 | 1,00E-12 | 9,00E-05 | 5,1E-04 | 1,1 | 1,2 | 26 | 29 |
| S2 | 1,00E-13 | 1,00E-12 | 9,00E-05 | 1,2E-04 | 1,1 | 1,2 | 22 | 24 |
| S3 | 1,00E-13 | 1,00E-12 | 9,00E-05 | 1,5E-04 | 1,1 | 1,2 | 21 | 23 |
| S4 | 1,00E-13 | 1,00E-12 | 9,00E-05 | 3,0E-04 | 1,1 | 1,2 | 17 | 19 |
| S5 | 1,00E-13 | 1,00E-12 | 9,00E-05 | 1,1E-04 | 1,1 | 1,2 | 24 | 27 |
| S6 | 1,00E-13 | 1,00E-12 | 9,00E-05 | 2,0E-04 | 1,1 | 1,2 | 35 | 37 |
| S7 | 1,00E-13 | 1,00E-12 | 9,00E-05 | 1,4E-04 | 1,1 | 1,2 | 23 | 26 |
| S8 | 1,00E-13 | 1,00E-12 | 9,00E-05 | 5,7E-04 | 1,1 | 1,2 | 33 | 36 |
| S9 | 1,00E-13 | 1,00E-12 | 9,00E-05 | 5,7E-04 | 1,1 | 1,2 | 35 | 37 |

**Planckian distribution**

From the size principle section we know that the MU transitions can be seen as modes with energy differences between consecutive states given by $h\nu$, where the frequency $\nu$ characterizes the mode. Let us assume that there are a lot of these modes, and that the probability of occupying an energy level $E$ is $P(E)=Ae^{-E/bdT}$, where $dT=T_{M}-T_{M0}$, as before, $b$ and A are constants. The function $P(E)$is known as Boltzmann distribution. Some MUs will be at the bottom of some reference energy state; some will be in the next one, and so forth. What is the average energy per mode? To find the answer, we have to calculate the total energy of all the MUs and divide the result by the total number of these MUs belonging to that mode. Thus, we let $N_{0}$ be the number of MUs that are in the ground state (the lowest frequency state); $N_{1}$ the number of MUs in the state $E_{1}$; $N_{2}$ the number of MUs in the state $E_{2}$; and so on. We shall assume that the number $N_{1}$ that are in the first state will be the number $N_{0}$ that are in the ground state, times $e^{\frac{-h\nu}{bdT}}$. Similarly, $N_{2}$, the number of MUs in the second state, is $N_{2}=N_{1}e^{\frac{-h\nu}{bdT}}$. Let us call $e^{\frac{-h\nu}{bdT}}=x$. Then we simply have $N_{1}=N_{0}x$, $N_{2}=N_{0}x^{2}$,..$N_{n}=N_{0}x^{n}$. If a mode is in the ground state, there is no energy. If it is in the first state, the energy is $h\nu$ and there are $N_{1}$ MUs. So $N_{1}h\nu$ or $N_{0}xh\nu$ is how much energy we get from those. Those that are in the second state have $2h\nu$, and there are $N_{2}$ of them, so $N_{2}2h\nu=N_{0}x^{2}2h\nu$ is how much energy we get, and so on. Then we add it all together to get $E_{total}=N_{0}h\nu\left(0+x+2x^{2}+3x^{3}+.....\right)$. Now how many MUs are there in that mode? Of course, $N_{0}$ is the number that are in the ground state, $N_{1}$ in the first state, and so on, and we add them together, so: $N_{total}=N_{0}(1+x+x^{2}+x^{3}+...)$. Thus the average energy is $\left\langle E_{total}\right\rangle=N_{0}h\nu(0+x+2x^{2}+3x^{3}+...)/N_{0}(1+x+x^{2}+x^{3}...)$. If we call $y=x+2x^{2}+3x^{3}....$, then $yx=x^{2}+2x^{3}+3x^{4}+...$, and $y-yx=x+x^{2}+x^{3}+...$. Now, if $k=1+x+x^{2}+x^{3}+...$, then $kx=x+x^{2}+x^{3}+...$ and $kx=k-1$, thus $k=1/\left(1-x\right)$. But $y-yx=k-1$ or $y=\left(k-1\right)/\left(1-x\right)$ and substituting the expression for $k$, we obtain $y=x/\left(1-x\right)^{2}$. Finally $\left\langle E_{total}\right\rangle=h\nu y/k=h\nu/(x^{-1}-1)$ or $h\nu/\left(e^{\frac{\nu}{\nu_{0}}}-1\right)$, where $\nu_{0}=adT$. Now, how many modes per frequency unit in a cavity of volume V are there? Let us assume that the modes inside the volume $V$form a system a system of standing waves that mathematically can be describe by $R=R_{0}\sin\left(k_{x}x\right)\sin\left(k_{y}y\right)\sin(k_{z}z)$ in each of the x,y,z directions. Since in a standing wave the amplitude at the walls must be zero, this implies that for each direction we must have that $k_{i}=n_{i}\pi/L$, where L is the length of the cavity in that direction, $n_{i}$ is an integer and since $k_{i}=2\pi/\lambda$, where $\lambda$ is the standing wave wavelength. Without loss of generality we can assume that $V=L^{3}$, and that $n_{x}=n_{y}=n_{z}=n$. Therefore the standing wave condition reads as $n=2L/\lambda$. We need to evaluate the number of modes, which can meet this condition. An approximation can be made by assuming that the number of modes in the "n" space is given by the volume of a sphere or radius "n". In fact due to the sphere symmetry we only need to count one eighth of the total volume, that is the number $N$ of modes we are looking for is $N=4\pi n^{3}/3x8=4\pi8L^{3}/\lambda^{3}3x8$ or $N=4\pi L^{3}/3\lambda^{3}$. We can rewrite $N$ in terms of the frequency by using the relationship $\nu=c_{m}/\lambda$, where $c_{m}$ is the conduction velocity of the muscle fiber, to obtain $N=4\pi L^{3}\nu^{3}/3c_{m}^{3}$. The number of modes per unit frequency, assuming that the there is only one possible polarization for the modes, would be given by $dN/d\nu=4\pi L^{3}\nu^{2}/c_{m}^{3}=4\pi V\nu^{2}/c_{m}^{3}$ and we can calculate the energy per unit frequency as $E(\nu,\nu_{0})=\left\langle E_{total}\right\rangle dN/d\nu$. In order to calculate the radiated energy per unit frequency, if we consider first that the energy is radiated perpendicularly to a small increment of area, then half of the energy is going towards the walls of the cavity and half is coming out if the system is in thermal equilibrium, that is, $P\left(\nu,\nu_{0}\right)=0.5dE\left(\nu,\nu_{0}\right)/dt$ or$P\left(\nu,\nu_{0}\right)=0.5u(\nu,\nu_{0})dV/dt$, where $u(\nu,\nu_{0})$ is the energy density per unit frequency given by $u(\nu,\nu_{0})=\left\langle E_{total}\right\rangle(dN/d\nu)/V$. At equilibrium $dV/dt=Sdx/dt=Sc_{m}$, where $S$ is the emission surface area and $x$ represents the direction where the energy is being radiated. Therefore $P(\nu,\nu_{0})=0.5u\left(\nu,\nu_{0}\right)Sc_{m}$ but at an angle $\theta$, the area changes to the effective area $S\cos\left(\theta\right)$ and $dx/dt$ changes to $\cos(\theta)(dx/dt)=c_{m}\cos(\theta)$ thus in general $P(\nu,\nu_{0},\theta)=0.5u\left(\nu,\nu_{0}\right)Sc_{m}\cos(\theta)^{2}$. Thus we can average for all the angles to obtain $P(\nu,\nu_{0})=\int_{0}^{\pi} 0.5u\left(\nu,\nu_{0}\right)Sc_{m}\cos(\theta)^{2}d\theta/\pi$, and finally $P(\nu,\nu_{0})=0.25u(\nu,\nu_{0})Sc_{m}$or $P(\nu,\nu_{0})=0.25(h\nu/(e^{\frac{\nu}{\nu_{0}}}-1))(4\pi\nu^{2}/c_{m}^{3})Sc_{m}$, and working out all the terms we obtain $P\left(\nu,\nu_{0}\right)=\frac{Sh\pi}{c_{m}^{2}}\left(\frac{\nu^{3}}{e^{\frac{\nu}{\nu_{0}}}-1}\right)$ as we wanted to show.

1. Merletti R, Sabbahi MA, De Luca CJ (1984) Median frequency of the myoelectric signal : Effects of muscle ischemia and cooling. European Journal of Applied Physiology 52: 258-265.

2. Basmajian JV, De Luca CJ (1985) Introduction. In: Butler J, editor. Muscles Alive Their functions revealed by Electromyography. Fifth ed. Baltimore: Willams & Wilkins. pp. 10-18.

3. Lindstrom LH, Magnusson RI (1977) Interpretation of Myoelectric Power Spectra : A model and it applications. Proceedings of the IEEE 65: 653-662.

4. Ward MR (1971) Electrical Engineering Science: McGraw-Hill.

5. Conwit RA, Stashuk D, Tracy B, McHugh M, Brown WF, et al. (1999) The relationship of motor unit size, firing rate and force. Clinical Neurophysiology 110: 1270-1275.

6. Monster AW, Chan H (1977) Isometric Force Production by Motor Units of Extensor Digitorum Communis Muscle in Man. J Neurophysiol 40: 1432-1443.

7. Zubieta-Calleja G, Pauley PE (2004) Metabolism. New Human Physiology. Copenhagen.

8. Nocedal J, Wright S (2004) Numerical Optimization: Springer.

S1 Fig. The relationship of versus values. The slope value is V^2^/Hz^2^. The error bars represent the propagation of uncertainty via algebraic manipulations of the individual standard errors in the firing frequency and amplitude for each force level from data presented in [5].
